# Supplementary material for: Effects of implementing non-nutritive sucking on oral feeding progression and outcomes in preterm infants: A systematic review and meta-analysis
Source: PLoS One. 2024 Apr 16;19(4):e0302267. doi: 10.1371/journal.pone.0302267 (PMC11020483; doi:10.1371/journal.pone.0302267)
Supplement: S1 Table — (DOCX) [file pone.0302267.s002.docx]

**Supplementary Table1. Search strategy.**

1. **Search criteria for PubMed:**

**#1** (( "Sucking Behavior"[Mesh] ) OR "Pacifiers"[Mesh]) OR ((non-nutritive AND suck*) OR (non-nutritive sucking) OR（Non-nutritional sucking） OR pacifier OR dummy OR soother OR nipple ) [All Fields]

**#2** (("Infant, Newborn"[Mesh]) OR "Infant, Premature"[Mesh]) OR (newborn OR neonate OR neonatal OR premature OR low birth weight OR VLBW OR LBW) [All Fields]

**#3** (randomized controlled trial OR controlled clinical trial [Publication Type])

**#4** #1 AND #2 AND #3 **367**

1. **Search criteria for Embase:**

**#1**'pacifier'/exp OR 'sucking'/exp OR ('non nutritive' AND suck* OR ('non nutritive' AND sucking)) AND 'or（non nutritional' AND sucking） OR pacifier OR dummy OR soother OR nipple

**#2**'prematurity'/exp OR ((newborn OR neonate OR neonatal OR premature OR low) AND birth AND weight) OR vlbw OR 'low birth weight' OR 'newborn'/exp

**#3** 'randomized controlled trial' OR 'controlled clinical trial'

**#4** #1 AND #2 AND #3  **339**

**C) Search criteria for Web of Science:**

**#1** TS=("Sucking Behavior" OR "Pacifiers"OR (non-nutritive AND suck*) OR (non-nutritive sucking) OR（Non-nutritional sucking） OR pacifier OR dummy OR smoother OR nipple

**#2** TS=("Infant, Newborn" OR "Infant, Premature" OR newborn OR neonate OR neonatal OR premature OR low birth weight OR VLBW OR LBW)

**#3** ALL=(randomized controlled trial OR controlled clinical trial)

**#4** #1 AND #2 AND #3  **177**

**C) Search criteria for CINAHL:**

**#1** SU=("Sucking Behavior" OR "Pacifiers"OR (non-nutritive AND suck*) OR (non-nutritive sucking) OR（Non-nutritional sucking） OR pacifier OR dummy OR smoother OR nipple

**#2** SU=("Infant, Newborn" OR "Infant, Premature" OR newborn OR neonate OR neonatal OR premature OR low birth weight OR VLBW OR LBW)

**#3** #1 AND #2  **169**

**D) Search criteria for the Cochrane Library:**

**#1** ( "Infant, Newborn" OR "Infant, Premature" OR newborn OR neonate OR neonatal OR premature OR low birth weight OR VLBW OR LBW ):ti,ab,kw

**#2**(randomized controlled trial OR controlled clinical trial):ti,ab,kw

**#3** (*"Sucking Behavior" OR "Pacifiers"OR (non-nutritive AND suck*) OR (non-nutritive sucking) OR（Non-nutritional sucking） OR pacifier OR dummy OR smoother OR nipple):ti,ab,kw

**#4** (oral inflation) OR (oral feeding):ti,ab,kw

**#5** #1AND #2 AND #3 AND#4  **671**

**E) Search criteria for CNKI:**

**#1** ("Sucking Behavior" OR "Pacifiers"OR (non-nutritive AND suck*) OR (non-nutritive sucking) OR（Non-nutritional sucking）OR pacifier OR dummy OR smoother OR nipple

**#2** ("Infant, Newborn" OR "Infant, Premature" OR newborn OR neonate OR neonatal OR premature OR low birth weight OR VLBW OR LBW)

**#3** (oral inflation) OR (oral feeding) OR (Preterm Infant Feeding)

**#4** #1 AND #2 AND #3  **349**

**F) Search criteria for the Wanfang:**

**#1** ("Sucking Behavior" OR "Pacifiers"OR (non-nutritive AND suck*) OR (non-nutritive sucking) OR（Non-nutritional sucking） OR pacifier OR dummy OR smoother OR nipple

**#2** ("Infant, Newborn" OR "Infant, Premature" OR newborn OR neonate OR neonatal OR premature OR low birth weight OR VLBW OR LBW)

**#3** (oral inflation) OR (oral feeding) OR (Preterm Infant Feeding)

**#4** #1 AND #2 AND #3 708

**G) Search criteria for VIP:**

**#1** ("Sucking Behavior" OR "Pacifiers"OR (non-nutritive AND suck*) OR (non-nutritive sucking) OR（Non-nutritional sucking） OR pacifier OR dummy OR smoother OR nipple

**#2** ("Infant, Newborn" OR "Infant, Premature" OR newborn OR neonate OR neonatal OR premature OR low birth weight OR VLBW OR LBW)

**#3** (oral inflation) OR (oral feeding) OR (Preterm Infant Feeding)

**#4** #1 AND #2 AND #3 **458**
