# Supplementary figures and images for: Effects of implementing non-nutritive sucking on oral feeding progression and outcomes in preterm infants: A systematic review and meta-analysis
Source: PLoS One. 2024 Apr 16;19(4):e0302267. doi: 10.1371/journal.pone.0302267 (PMC11020483; doi:10.1371/journal.pone.0302267)

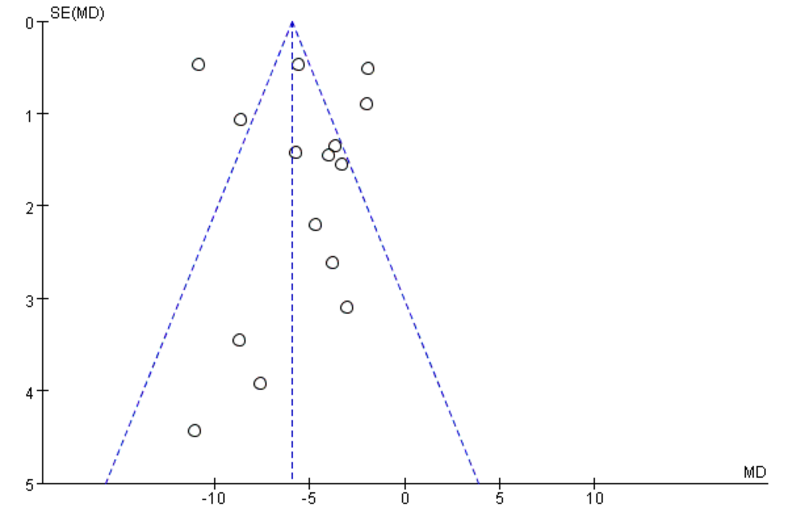

Supplement: S1 Fig — ((a): Time taken to achieve exclusive oral feeding; (b): Length of hospital stay; (c): Time to start oral feeding). (ZIP) [file pone.0302267.s003.zip › S1Fig (a).tif]

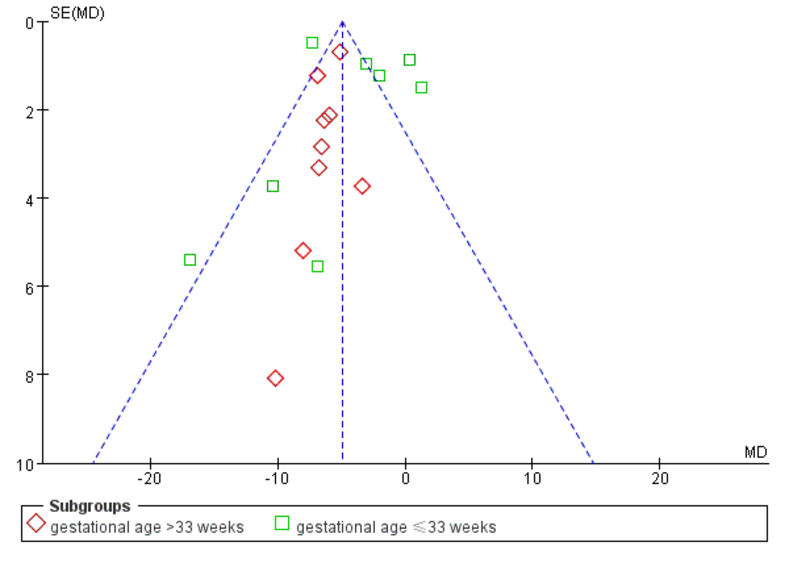

Supplement: S1 Fig — ((a): Time taken to achieve exclusive oral feeding; (b): Length of hospital stay; (c): Time to start oral feeding). (ZIP) [file pone.0302267.s003.zip › S1Fig (b).tif]

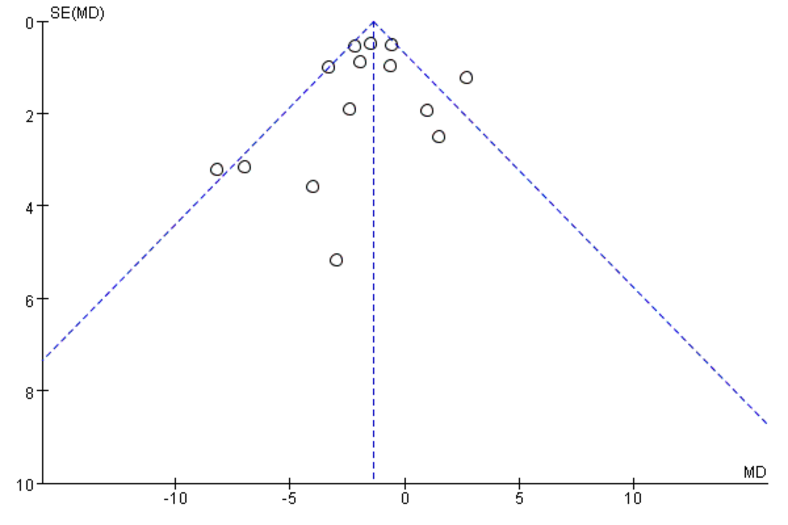

Supplement: S1 Fig — ((a): Time taken to achieve exclusive oral feeding; (b): Length of hospital stay; (c): Time to start oral feeding). (ZIP) [file pone.0302267.s003.zip › S1Fig (c).tif]
