# Supplementary material for: Effects of implementing non-nutritive sucking on oral feeding progression and outcomes in preterm infants: A systematic review and meta-analysis
Source: PLoS One. 2024 Apr 16;19(4):e0302267. doi: 10.1371/journal.pone.0302267 (PMC11020483; doi:10.1371/journal.pone.0302267)
Supplement: Attachment — Submitted filename: PONE-D-23-30243_SM.pdf [file pone.0302267.s004.pdf]

PLOS ONE

Effectiveness of non-nutritive sucking on oral feeding of preterm infants: a systematic review and meta-analysis

--Manuscript Draft--

**Manuscript Number:**	PONE-D-23-30243
**Article Type:**	Research Article
**Full Title:**	Effectiveness of non-nutritive sucking on oral feeding of preterm infants: a systematic review and meta-analysis
**Short Title:**	Effectiveness of non-nutritive sucking on oral feeding of preterm infants
**Corresponding Author:**	xinghui cui<br>Weifang Medical University - Fuyanshan Campus: Weifang Medical University<br>weifang, shangdong CHINA
**Keywords:**	non-nutritive sucking; oral feeding; preterm infants; premature; meta-analysis
**Abstract:**	Background<br>Preterm infants have imperfect neurological development, uncoordinated sucking-swallowing-breathing, which makes it difficult to realize effective oral feeding after birth. How to help preterm infants achieve complete oral feeding as soon as possible has become an important issue in the management of preterm infants. Non-nutritive sucking (NNS), as a useful oral stimulation, can improve the effect of oral feeding in preterm infants.<br>Methods<br>Our study was registered in PROSPERO (CRD42023457646) prior to its commencement. We systematically searched for randomized controlled trials (RCTs) published before August 2023 in PubMed, Web of Science, Embase, Cochrane databases, China's National Knowledge Infrastructure, Wanfang and VIP database.<br>Results<br>A total of 21 randomized controlled trials with 1443 preterm infants were included. The results of the meta-analysis showed that NNS significantly shortened time taken to achieve exclusive oral feeding (MD=-4.65,95%CI=-6.11to-3.20, p < 0.001), length of hospital stay(MD=-4.58, 95% CI= -6.40 to -2.76, p < 0.001), time to start oral feeding(MD=-1.41, 95% CI= -2.36 to -0.45, p =0.004), time to return to birth weight(MD= -1.72, 95% CI= -2.54 to -0.91, p < 0.001). Compared to the NNS group, the control group had significant weight gain in preterm infants, including weight of discharge (MD= -63.48, 95% CI= -97.93 to -29.03, p =0.0003), weight at full oral feeding (MD= -92.91, 95% CI= -142.68 to -43.13, p =0.0003). In addition, NNS reduced the incidence of gastrointestinal dysfunction complications(OR= 0.22, 95% CI= 0.14 to 0.35, p < 0.001) in preterm infants.<br>Conclusion<br>In summary, NNS improves oral feeding outcomes in preterm infants and reduces oral feeding and hospitalization length. More studies are needed to validate the effects on weight gain and growth in preterm infants. However, we believe that the results of our meta-analysis will help to update the existing evidence on NNS to improve oral feeding in preterm infants and provide a basis for promoting rational feeding in preterm infants.
**Order of Authors:**	Shuliang Zhao
	Huimin Jiang
	Yiqun Miao
	Wenwen Liu
	Yanan Li
	Yuanyuan Zhang
	Aihua wang
	Xinghui Cui
**Additional Information:**	

Powered by Editorial Manager® and ProduXion Manager® from Aries Systems Corporation

Question	Response
**Financial Disclosure**<br><br>Enter a financial disclosure statement that describes the sources of funding for the work included in this submission. Review the submission guidelines for detailed requirements. View published research articles from *PLOS ONE* for specific examples.<br><br>This statement is required for submission and **will appear in the published article** if the submission is accepted. Please make sure it is accurate.<br><br>**Unfunded studies**<br>Enter: *The author(s) received no specific funding for this work.*<br><br>**Funded studies**<br>Enter a statement with the following details:<br>• Initials of the authors who received each award<br>• Grant numbers awarded to each author<br>• The full name of each funder<br>• URL of each funder website<br>• Did the sponsors or funders play any role in the study design, data collection and analysis, decision to publish, or preparation of the manuscript?<br>• **NO** - Include this sentence at the end of your statement: *The funders had no role in study design, data collection and analysis, decision to publish, or preparation of the manuscript.*<br>• **YES** - Specify the role(s) played.<br><br>* typeset	The author(s) received no specific funding for this work.
**Competing Interests**<br><br>Use the instructions below to enter a competing interest statement for this submission. On behalf of all authors, disclose any competing interests that could be perceived to bias this work—acknowledging all financial support and any other relevant financial or non-financial competing interests.	The authors have declared that no competing interests exist.

This statement is required for submission and **will appear in the published article** if the submission is accepted. Please make sure it is accurate and that any funding sources listed in your Funding Information later in the submission form are also declared in your Financial Disclosure statement.<br><br>View published research articles from *PLOS ONE* for specific examples.<br><br>**NO authors have competing interests**<br><br>Enter: *The authors have declared that no competing interests exist.*<br><br>**Authors with competing interests**<br><br>Enter competing interest details beginning with this statement:<br><br>*I have read the journal's policy and the authors of this manuscript have the following competing interests: [insert competing interests here]*<br><br>* typeset	
**Ethics Statement**<br><br>Enter an ethics statement for this submission. This statement is required if the study involved:<br><br>• Human participants<br>• Human specimens or tissue<br>• Vertebrate animals or cephalopods<br>• Vertebrate embryos or tissues<br>• Field research<br><br>Write "N/A" if the submission does not require an ethics statement.<br><br>General guidance is provided below. Consult the submission guidelines for detailed instructions. **Make sure that all information entered here is included in the Methods section of the manuscript.**	"N/A"

Powered by Editorial Manager® and ProduXion Manager® from Aries Systems Corporation

**Format for specific study types**<br><br>**Human Subject Research (involving human participants and/or tissue)**<br>• Give the name of the institutional review board or ethics committee that approved the study<br>• Include the approval number and/or a statement indicating approval of this research<br>• Indicate the form of consent obtained (written/oral) or the reason that consent was not obtained (e.g. the data were analyzed anonymously)<br><br>**Animal Research (involving vertebrate animals, embryos or tissues)**<br>• Provide the name of the Institutional Animal Care and Use Committee (IACUC) or other relevant ethics board that reviewed the study protocol, and indicate whether they approved this research or granted a formal waiver of ethical approval<br>• Include an approval number if one was obtained<br>• If the study involved *non-human primates*, add *additional details* about animal welfare and steps taken to ameliorate suffering<br>• If anesthesia, euthanasia, or any kind of animal sacrifice is part of the study, include briefly which substances and/or methods were applied<br><br>**Field Research**<br><br>Include the following details if this study involves the collection of plant, animal, or other materials from a natural setting:<br>• Field permit number<br>• Name of the institution or relevant body that granted permission	
**Data Availability**<br><br>Authors are required to make all data underlying the findings described fully available, without restriction, and from the time of publication. PLOS allows rare exceptions to address legal and ethical concerns. See the PLOS Data Policy and FAQ for detailed information.	No - some restrictions will apply

A Data Availability Statement describing where the data can be found is required at submission. Your answers to this question constitute the Data Availability Statement and **will be published in the article**, if accepted.<br><br>**Important:** Stating 'data available on request from the author' is not sufficient. If your data are only available upon request, select 'No' for the first question and explain your exceptional situation in the text box.<br><br>Do the authors confirm that all data underlying the findings described in their manuscript are fully available without restriction?	
**Describe where the data may be found in full sentences. If you are copying our sample text, replace any instances of XXX with the appropriate details.**<br><br>• If the data are **held or will be held in a public repository**, include URLs, accession numbers or DOIs. If this information will only be available after acceptance, indicate this by ticking the box below. For example: *All XXX files are available from the XXX database (accession number(s) XXX, XXX.*).<br>• If the data are all contained **within the manuscript and/or Supporting Information files**, enter the following: *All relevant data are within the manuscript and its Supporting Information files.*<br>• If neither of these applies but you are able to provide **details of access elsewhere**, with or without limitations, please do so. For example:<br><br>*Data cannot be shared publicly because of [XXX]. Data are available from the XXX Institutional Data Access / Ethics Committee (contact via XXX) for researchers who meet the criteria for access to confidential data.*<br><br>*The data underlying the results presented in the study are available*	All relevant data are within the manuscript and its Supporting Information files.

from (include the name of the third party and contact information or URL).<br>• This text is appropriate if the data are owned by a third party and authors do not have permission to share the data.<br><br>* typeset	
Additional data availability information:	Tick here if your circumstances are not covered by the questions above and you need the journal’s help to make your data available.

manuscript Click here to access/download;Manuscript;manuscript.docx

Effectiveness of non-nutritive sucking on oral feeding of preterm infants: a systematic review and meta-analysis

Shuliang Zhao[1,2,+] , Huimin Jiang[1,+] , Yiqun Miao[2] ,Wenwen Liu[3] ,Yanan Li[1] , Yuanyuan Zhang[1] , Aihua Wang[1*], Xinghui Cui[4*]

[1] School of Nursing, Weifang Medical University, Weifang 261053, China

[2] School of Nursing, Capital Medical University, Beijing 100071, China

[3] Xiangya School of Nursing, Central South University, Changsha 410000, China;

[4] Nursing Department Affiliated Hospital of Weifang Medical University, Weifang 261031, China

+ These authors contributed equally to this work and co-first authors.

*Corresponding author: Aihua Wang (Wangaihua64@163.com) and Xinghui Cui (lcyxycxh@163.com)

ORCID: 0000000259618097(AW); 0000000259618097 (XC)

Effectiveness of non-nutritive sucking on oral feeding of preterm infants: a systematic review and meta-analysis

Abstract

Background

Preterm infants have imperfect neurological development, uncoordinated sucking-swallowing-breathing, which makes it difficult to realize effective oral feeding after birth. How to help preterm infants achieve complete oral feeding as soon as possible has become an important issue in the management of preterm infants. Non-nutritive sucking (NNS), as a useful oral stimulation, can improve the effect of oral feeding in preterm infants.

Methods

Our study was registered in PROSPERO (CRD42023457646) prior to its commencement. We systematically searched for randomized controlled trials (RCTs) published before August 2023 in PubMed, Web of Science, Embase, Cochrane databases, China's National Knowledge Infrastructure, Wanfang and VIP database.

Results

A total of 21 randomized controlled trials with 1443 preterm infants were included. The results of the meta-analysis showed that NNS significantly shortened time taken to achieve exclusive oral feeding (MD=-4.65,95%CI=-6.11to-3.20, $p < 0.001$), length of hospital stay(MD=-4.58, 95% CI= -6.40 to -2.76, $p < 0.001$), time to start oral feeding(MD=-1.41, 95% CI= -2.36 to -0.45, p =0.004), time to return to birth

weight(MD= -1.72, 95% CI= -2.54 to -0.91, $p < 0.001$). Compared to the NNS group, the control group had significant weight gain in preterm infants, including weight of discharge (MD= -63.48, 95% CI= -97.93 to -29.03, p =0.0003), weight at full oral feeding (MD= -92.91, 95% CI= -142.68 to -43.13, p =0.0003). In addition, NNS reduced the incidence of gastrointestinal dysfunction complications(OR= 0.22, 95% CI= 0.14 to 0.35, $p < 0.001$) in preterm infants.

Conclusion

In summary, NNS improves oral feeding outcomes in preterm infants and reduces oral feeding and hospitalization length. More studies are needed to validate the effects on weight gain and growth in preterm infants. However, we believe that the results of our meta-analysis will help to update the existing evidence on NNS to improve oral feeding in preterm infants and provide a basis for promoting rational feeding in preterm infants.

Keywords non-nutritive sucking; oral feeding; preterm infants; premature; meta-analysis

Introduction

Currently, oral feeding and swallowing problems in preterm infants are common medical problems in neonatal intensive care units (NICUs) and families of preterm infant [1]. Due to the small birth gestational age of preterm infants, the cardiopulmonary system, central nervous system and oral musculature are not yet fully developed [2], and the lack of mechanisms to control oral movements, as well as poor coordination of sucking, swallowing and respiration, can easily lead to oral feeding difficulties in preterm infants [3, 4]. The transition from tube-feeding to full oral feeding is a ~~huge~~

challenge for infant caregivers [5]. Early difficulties with oral feeding can affect the ability of preterm infants to achieve independent oral feeding, prolong hospitalization, and cause long-term feeding problems such as malnutrition, growth retardation, and cognitive deficits [2, 6, 7]. Therefore, providing optimal nutritional feeding support for preterm infants, improving sucking and feeding behaviors in infants less than 30 weeks of age, and increasing the ability to feed independently through the mouth are key to the early care and survival of preterm infants [8].

Non-nutritive sucking (NNS) and nutritive sucking (NS) are used as indicators of an infant's oral motion and behavior [9]. NNS refers to the process that premature infants who can't accept oral feeding suck a non-porous pacifier without milk intake after stimulating the oral lip of premature infants before feeding [10]. NNS creates an oral feeding experience ~~without increasing fluid pressure and is one of the early~~ and promotes ~~methods of infant~~ self-organization and ~~self-~~soothing. ~~As an essential skill in infancy, NNS is important for oral feeding and self-regulation~~. Although there seems to be a relationship between NNS and oral feeding, the ability of NNS does not necessarily indicate the success of feeding [9]. Oral feeding not only necessitates the components of NNS but also requires suck–swallow–breathe coordination to manage a bolus for consumption [11].

Boiron et al. [12] state that “NNS promotes the coordination of sucking and swallowing, accelerates the maturation of the sucking reflex，improves the initiation and duration of the first nutritive sucking.” Previous Cochrane analyses [13-16] have shown that NNS increased physiologic stability and nutrition in preterm infants and

reduced the length of hospitalization, but it did not reach a consistent conclusion on the weight gain of premature infants. Although a few published meta-analyses have reached a consensu[illegible]n recent years, additional randomized controlled trials have been conducted, so we decided to carry out a more comprehensive and systematic literature review and meta-analysis to compar[illegible]e effect of NNS on oral feeding in preterm infants and to add secondary outcome indicators such as discharge weight, weight at full oral feeding, time to return to birth weight, and gastrointestinal complications in both groups. Our meta-analysis was to explor[illegible]e effect of using NNS on the smooth transition to oral feeding in preterm infants and to provide the latest evidence for the clinical use of NNS.

Materials and methods

Systematic search and strategy

The review protocol was developed using The Cochrane Handbook for Systematic Reviews of Interventions. Our meta-analysis was conducted according to the preferred reporting items in the guide of systematic review and meta-analysis (PRISMA) [17]. The PRISMA checklist is presented in S1Table[illegible]e searched PubMed, Web of science, Embase, Cochrane Library, CNKI, VIP and Wanfang databases were conducted for randomized controlled trials from creation to August 1, 2023. The following search terms were used:("Sucking Behavior" OR "Pacifiers" OR (non-nutritive AND suck*) OR (non-nutritive sucking) OR (Non-nutritional sucking) OR pacifier OR dummy OR soother OR nipple) AND ("Infant, Newborn" OR "Infant, Premature" OR newborn OR neonate OR neonatal OR premature OR low birth weight O[illegible]LBW OR LBW[illegible]ND

(randomized controlled trial OR controlled clinical trial OR RCT). Detailed search strategies for each database are shown in S2 Table. Only research involving human beings is included in the selection. Moreover, language limitations are English or Chinese. Our meta-analysis was registered with the PROSPERO database (CRD42023457646).

Inclusion and exclusion criteria

Studies included in this meta-analysis must meet the following inclusion criteria: ①Preterm infants with gestational age <37 weeks and birth weight <2500g; ②Randomized controlled studies; ③Preterm infants in the intervention group received NNS (The intervention can occur before, during or after gavage feeding. NNS involving the use of a pacifier or nipple.) and preterm infants in the control group were implemented on routine feeding. ④ At least one of the parameters was included in the outcome measures: Time taken to achieve exclusive oral feeding, Length of hospital stay, Time to start oral feeding, Weight of discharge, Weight at full oral feeding, Time to return to birth weight, Gastrointestinal complications. Time taken to achieve exclusive oral feeding was defined as when the infant ingests all nutrient volumes in a 24hour period without any gavage. Length of hospitalization was described as the number of days from admission to hospital discharge. Time to start oral feeding was defined as transition from gavage feeding to oral feeding.

please add the defintion for remaining outcomes and specify the unit of measures weight at discharge ,weight at full oral feeding, time to return to birth weight and GI complications

Any study that met the exclusion criteria was excluded from the meta-analysis: ①Reviews, case reports, observational studies or animal trials. ②Full text was unavailable ③Studies with insufficient information for extraction data ④Unclear or

inappropriate definition of exposure/results ⑤NNS was implemented in the control group.

Data extraction

Two researchers (ZSL and JHM) screened through the literature independently and completed the extraction of data. If there is a disagreement, a third researcher (MYQ) should be called in to adjudicate. Initial screening was done first by reading the title and abstract, followed by selecting the final compliant literature by reading the full text. The extracted information includes the following aspects: first author's name, year of publication, country, sample size, details of the intervention, and the data of outcome indicators. If the units of the outcome indicators in the study are inconsistent, they should be converted to harmonized units prior to subsequent processing.

Methodological quality assessment

We assessed the methodological quality of RCTs using the Cochrane risk of bias tool [18], which included seven evaluation items: sequence generation, allocation concealment, blinding of participants, outcome evaluator, incomplete outcome data, and selective outcome reporting. The studies were evaluated for unclear, low or high risk of bias. In addition, we entered the assessment data into Cochrane Review Manager software (Revman version 5.3, Copenhagen: The Nordic Cochrane Centre, The Cochrane Collaboration, Denmark) to obtain a risk of bias assessment table. This was done independently by two assessors(LWW and LYN), and when disagreements arose, they were resolved in consultation with a third researcher (ZSL)until agreement was reached.

Data synthesis and analysis

The data was analyzed by Review Manager 5.3 software. The mean difference (MD) and confidence interval (CI) of 95% were calculated for continuous data, while the pooled odds ratios (ORs) and confidence interval (CI) of 95% were estimated for dichotomous data. Heterogeneity was assessed by using I^2 statistics. $I^2 \geq 50\%$ indicated significant heterogeneity between studies, and the random effect model was used for analysis. $I^2 < 50\%$, considered little heterogeneity, fixed effects model used. Sensitivity analysis was conducted by changing the analytical model, i.e., selecting the opposite model according to the original analytical model and conducting the combined analysis of effect sizes again. If the results of the two models were consistent, it indicated that the sensitivity was lower, suggesting that the results of the meta-analysis were stable, and vice versa, suggesting that the sensitivity was higher and the results of the meta-analysis were unstable. After individual studies were sequentially excluded, the data were reanalyzed and sensitivity analyses were performed by comparing the excluded results with the original results. Subgroup analyses were used when necessary to address heterogeneity. Funnel plot was performed to evaluate publication bias. $P < 0.05$ was considered to be statistically significant.

Results

Search results

The initial search found 2986 studies, 1268 of which were duplicated. After analyzing the titles and abstracts, 68 studies were eligible for full-text screening. A total of 21 studies with 1443 preterm infants were finally included, 777 in the intervention group

and 666 in the control group. The flowchart depicts the selection procedure for the 21 studies (Fig 1). Table 1 shows the detailed characteristics of the included literature. These articles included were published from 1982 to 2022. Ten studies [19-28] were conducted in China, three studies [29-31] in the United States, three studies [32-34] in Iran, three studies [35-37] in Turkey, and one each in Canada [38] and Brazil [39]. Fig 2 depicts the risk-of-bias assessment in minute detail. Only five studies showed a low risk of bias [28, 32-34, 39], and fifteen studies [19-27, 29-31, 35, 37, 38] showed an unclear risk of bias. Because it is difficult to implement double-blinding for NNS implementation, only five [28, 32-34, 39] of the included papers were better blinded.

Meta analysis results

Primary Outcomes

Time taken to achieve exclusive oral feeding. Time taken to achieve exclusive oral feeding(days) was included as an outcome between the two groups in fourteen studies [19-21, 25, 28, 29, 31-36, 38, 39] that involved 910 premature infants. We observed that the time to transition to full oral feeding in the NNS group was significantly shorter compared to the control group (MD=-4.65,95%CI=-6.11to-3.20, p ＜0.001) (Fig 3).Heterogeneity was reduced after two articles [20, 29] was removed from the sensitivity analysis ($I^2 = 36\%$, $p = 0.10$).

Length of hospital stay. Sixteen studies [19, 20, 22, 25, 29-31, 33-39] involving 974 premature infants examined the effect of NNS on length of hospital stay and contributed data to the meta-analysis. Meta-analysis showed a statistically significant shorter length of hospital stay for infants in the NNS compared to the control infants

(MD=-4.58, 95% CI= -6.40 to -2.76, $p < 0.001$). Significant heterogeneity existed among these studies (I^2 =75%, $p < 0.001$).Subgroup analyses was performed based on gestational age of preterm infants (Fig 4). Heterogeneity was significantly reduced in preterm infants with gestational age >33 weeks (I^2 =0%, p=0.94), and statistical differences between the NNS and control groups remained after grouping.

Time to start oral feeding. Fourteen studies [19-24, 26, 27, 29, 30, 32, 33, 37, 39] involving 953 premature infants included time to start oral feeding as an outcome. Compared to the control group , we found a statistically significant reduction in transition from gavage to start oral feeding in the NNS group(MD=-1.41, 95% CI= -2.36 to -0.45, p=0.004) (Fig 5), and there was greater heterogeneity(I^2 =59%, p=0.003). Heterogeneity was reduced after one article [29] was removed from the sensitivity analysis (I^2 =40%, p=0.07).

The Secondary Outcomes

Weight of discharge. Weight of discharge was included as an outcome between the two groups in seven studies [20, 28, 29, 35-37, 39] that involved 543 infants. Meta-analysis showed that there was a significant difference in weight at discharge between the two groups. Compared to the control group, preterm infants in the NNS group weighed less at hospital discharge than the control group (MD= -63.48, 95% CI= -97.93 to -29.03, p =0.0003) (Fig 6). Heterogeneity between studies was low (I^2 =3%, p=0.40), so a fixed-effects model was used.

Time to return to birth weight. Time to return to birth weight was included as an outcome between the two groups in seven studies [22-24, 26-28, 35] that involved

451 infants. Compared to the control group, preterm infants in the NNS group had a shorter time to return to birth weight (MD= -1.72, 95% CI= -2.54 to -0.91, $p < 0.001$) (Fig 7). Significant heterogeneity existed among these studies ($I^2 = 51\%$, $p = 0.06$), so the random-effect model was used.

Weight at full oral feeding. Five studies [19, 28, 37-39] involving 314 premature infants reported the weight at full oral feeding as an outcome. The results of meta-analysis showed that there was a significant difference between the two groups, with lighter preterm infants in the NNS group (MD= -92.91, 95% CI= -142.68 to -43.13, p =0.0003) (Fig 8). There was no significant heterogeneity between these studies (I^2 = 0%, $p = 0.95$), so a fixed-effects model was used.

Gastrointestinal complications. Seven studies [20-22, 24, 26, 27, 35] involving 549 premature infants reported the gastrointestinal complications as an outcome. According to our meta-analysis, gastrointestinal complications in preterm infants were significantly less in the NNS group than in the control group(OR= 0.22, 95% CI= 0.14 to 0.35, $p < 0.001$) (Fig 9). There was no significant heterogeneity between these studies ($I^2 = 0\%$, $p = 0.86$), so a fixed-effects model was used.

Sensitivity analysis and publication bias

Sensitivity analysis by converting the effect sizes of the fixed-effects model and the random-effects model to each other revealed that the differences in the combined effect sizes of the two were small, indicating that the results of the meta-analysis were generally stable (Table 2). In addition, the source of heterogeneity was further explored using a study-by-study approach to exclude individual studies. We drew funnel plots

for the indicators of the number of 10 or more articles included in the literature and evaluated the publication bias. The results showed that the funnel plots were all largely symmetrical, with no obvious publication bias (S1 Fig).

Discussion

Studies in recent years have shown that NNS not only enables nasogastric tube-fed preterm infants to transition to oral feeding as soon as possible, but also has a significant effect on both behavioral patterns and growth and development of preterm infants [40, 41]. It seems to be an undeniable fact that the use of pacifier or NNS affects the sucking skills of premature infants. However, NNS is not a method that must absolutely be used in every infant, and it is recommended in cases where the benefits are believed to outweigh the side effects [37][illegible] Considering the negative impact of NNS on infant health and the controversy surrounding soothers, the present study conducted a more comprehensive and systematic meta-analysis aimed at investigating the impact of NNS on the effectiveness of oral feeding in preterm infants. The NNS group was associated with shorter time required to achieve pure oral feeding, length of hospitalization, time to start oral feeding and time to regain birth weight compared to the control group. Preterm infants in the NNS group showed significant weight gain at hospital discharge and at complete oral feeding. In this review, evidence from multiple randomized trials supports that NNS prevents gastrointestinal-related complications in infants.

First of all, our meta-analysis found that the premature infants in NNS group had significantly shorter time taken to reach exclusive oral feeding and time to start oral feeding than those in the control group, which was consistent with the Cochrane

analysis of Foster et al. [13]. Preterm infant perioral and oral receptors are highly sensitive to stimulation, mechanical stimulation of the lips or tongue can cause a non-specific reflex in the orbicularis oris muscle, thus initiating sucking behavior [42]. The sensory experience generated by NNS can trigger sucking in preterm infants through the input-feedback mechanism of the central pattern generator (CPG), prompting an increase in the number of active sucks, a faster rate of sucking, an increase in the negative pressure of sucking, and a shortening of the stopping time, thus promoting the maturation of the sucking reflex [43]. In addition, NNS motor training of preterm infants' oral muscles promotes the coordination of sucking, swallowing, and respiration, thus accelerating the transition from tube-feeding to transoral feeding [40]. The meta-analysis by Tolppola et al. [44] (2022) was also in agreement with our findings, confirming the reduction in time from gavage to total mouth feeding in preterm infants in the NNS group, and suggesting that the use of NNS in hospitalized preterm infants is beneficial and should be carried out in clinical practice.

Adequate nutrition increases the rate of development of premature infant bodies, leading to shorter hospital stays. Consistent with the results of a previous meta-analysis [5, 16, 45], our study demonstrates that NNS significantly reduces the length of hospitalization for preterm infants. Say et al [35], Vailda[illegible]7]et al and Shaki et al [32] studies also showed that preterm infants in the NNS group were discharged earlier, and these results are consistent with the present study. Analyzing the reasons, this may be related to the fact that NNS not only promotes the maturation of sucking reflex in preterm infants and shortens the time from nasal feeding to complete oral feeding[46],

but also prompts hepatic, biliary and pancreatic activities through the vagus nerve, regulates gastrointestinal peptide levels, and stimulates gastrointestinal growth and development and maturation, which in turn improves feeding tolerance in preterm infants and shortens the length of hospital stay[23]. Clinical experience has demonstrated that the length of hospitalization of preterm infants is largely dependent on the time taken to achieve complete oral feeding. However, it has also been noted that the criteria for discharge of preterm infants is not only full oral feeding, but also related to many complex factors, such as the corrected gestational age of the preterm infant, weight, vital signs [47]. There should be good communication between hospitals, communities and families to safeguard the lives and health of preterm infants.

Our study showed that preterm infants in the NNS group had lower body weights at hospital discharge compared to the control group, a finding that is consistent with the findings of Lyu et al. [28]. To analyze the reasons, NNS shortened the duration of tube feeding for preterm infants, so that preterm infants reached complete transoral feeding earlier, therefore preterm infants in the NNS group were discharged earlier and weighed less at discharge. In addition, for preterm infants transoral feeding requires more energy consumption, which tends to cause fatigue and ultimately affects weight gain [28]. The studies of Asadollahpour et al. [34], Bernbaum et al. [31] and Calik et al. [48] contrasted with the results of the present study and concluded that NNS improved the weight of preterm infants at the time of discharge from the hospital, and that preterm infants in the NNS group weighed more heavily than those in the control group. The analysis may be related to the following factors: NNS can stimulate the vagus nerve excitation in the

oral cavity of preterm infants, and then stimulate the secretion of gastric motility and gastrin, regulate gastrointestinal motility, promote the development of gastrointestinal function, so that preterm infants eat more [49], but also cause vagus nerve excitation, accelerate the digestion and absorption of nutrients, and promote the growth of physical development [46]. In addition, NNS training can stimulate the orofacial and lingual muscles of preterm infants, improve the strength of oropharyngeal muscles, improve the function of swallowing reflexes, enhance the sucking ability of children, shorten the time of feeding through the mouth, and promote weight gain[50].Other studies[20, 35, 37] have also found that preterm infants in the NNS and control groups had similar weight measurements at discharge, and the difference was not statistically significant, but it does not indicate that NNS does not promote body mass growth in preterm infants. This is because there are many other factors involved in the weight gain and growth of preterm infants. The variability in the effect of NNS on discharge weight of preterm infants between studies may be related to the different methods of intervention, and there are differences in the timing as well as duration of NNS use that can affect the effectiveness of the intervention, so this result should be interpreted with caution.

Successful oral feeding has been defined as the ability of preterm infants to complete the prescribed amount of milk and gain the expected weight. However, our study found that the mean weight of preterm infants in the NNS group was lower than that of the control group at the time of achieving independent oral feeding, which is consistent with the findings of Lyu et al. [28]. In contrast, the findings of Rocha et al [39] found that NNS had no significant effect on the weight of preterm infants at the

time of independent mouth-feeding. The study by Rocha et al. [39] documented the average weekly weight gain, whereas most of the studies that we included measured the rate of weight gain over the entire period of hospitalization. Different measurements can affect the results of the study, in addition to the fact that the weight of preterm infants is influenced by other factors. The maximum degree of physiologic weight loss and the time to regain birth weight reflect nutrient availability and physical development. In our study, we found that the difference in time to regain birth weight between the two groups of preterm infants was statistically significant, which is consistent with the results of Zhang et al. [23] and Yue et al. [24]. NNS accelerates the maturation of the sucking reflex in preterm infants, improves the ability to suck, and facilitates oral gratification, which leads to an increase in the amount of milk ingested by the infants. In addition, the pituitary gland increased the secretion of various hormones, especially growth hormone secretion reached the highest level, which is conducive to the growth and development of preterm infants [51]. NNS can also enable infants to obtain ideal behavioral state, sedation and reduce provocation, make infants feel warm and safe, and reduce the consumption of energy [52].

In the present study, preterm infants in the NNS group presented (vomiting, reflux, abdominal distension) with significantly fewer gastrointestinal dysmotility complications than those in the control group, which is consistent with the findings of the Say et al. [35] and Feng et al. [21]. The possible explanation for this is that NNS helps to establish rhythmic sucking and swallowing patterns in preterm infants, promotes gastrointestinal growth and development and the maturity of gastrointestinal

function, and reduces feeding intolerance and gastrointestinal complications by stimulating the sensory nerve fibers in the oral cavity and stimulating the G-cells to release gastric motility, gastrin, and gastric acid secretion [53].

This review has the following limitations: First, although we conducted a relatively comprehensive search, we may have missed some eligible studies. Only articles written in English or Chinese were included, and gray literature was not considered. Second, some articles did not clearly describe randomization and blindness, which may lead to biased reports. Finally, there was some heterogeneity in the interventions, with some using a combination of oral stimulation and NNS, and some using only NNS, but as the results were similar across all studies, this should not have been an issue in the analysis.

Conclusions

In conclusion, NNS can significantly shorten the Time taken to achieve exclusive oral feeding, Length of hospital stay, Time to start oral feeding, Time to return to birth weight. We found that the weight of discharge, weight at full oral feeding of preterm infants in the NNS group was lower than that in the control group, but we cannot deny that NNS did not promote the weight gain of preterm infants. In the future, it is necessary to further explore the effect of NNS on weight gain of preterm infants, and to establish a standardized evidence-based management method for oral feeding of preterm infants. [illegible]t is worth noting that NNS also reduces the occurrence of complications related to gastrointestinal dysfunction, which facilitates the promotion of its use in NICU.

Supporting information

S1 Table. PRISMA 2020 checklist.

(DOCX)

S2 Table. Search strategy.

(DOCX)

S1 Fig. Funnel plot of publication bias. (S1Fig (a): Time taken to achieve exclusive oral feeding; S1Fig(b): Length of hospital stay; S1Fig(c): Time to start oral feeding.)

(ZIP)

Author Contributions

Conceptualization: Shuliang Zhao, Huimin Jiang, Yiqun Miao.

Data curation: Shuliang Zhao, Huimin Jiang, Wenwen Liu, Yanan Li.

Formal analysis: Shuliang Zhao, Huimin Jiang, Yiqun Miao.

Funding acquisition: Aihua Wang, Yuanyuan Zhang, Xinghui Cui.

Investigation: Shuliang Zhao, Huimin Jiang, Wenwen Liu, Yanan Li.

Methodology: Shuliang Zhao, Huimin Jiang, Yiqun Miao, Wenwen Liu, Yanan Li.

Project administration: Aihua Wang, Yuanyuan Zhang, Xinghui Cui.

Resources: Aihua Wang, Xinghui Cui.

Software: Shuliang Zhao, Huimin Jiang.

Validation: Shuliang Zhao, Yiqun Miao, Aihua Wang.

Writing – original draft: Shuliang Zhao, Huimin Jiang.

Writing – review & editing: Shuliang Zhao, Aihua Wang, Xinghui Cui.

References

1. Mousavi SZ, Shahbodaghi MR, Kadivar M, Khosravi N, Mirzazade M. The study of oral feeding disorders incidence in infants in NICU of Tehran University of medical sciences hospitals. Journal of Modern Rehabilitation. 2015;9:1-7.
2. Schneider N, Garcia-Rodenas CL. Early Nutritional Interventions for Brain and Cognitive Devel opment in Preterm Infants: A Review of the Literature. Nutrients. 2017;9(3). Epub 2017/03/01. do i: 10.3390/nu9030187. PubMed PMID: 28241501; PubMed Central PMCID: PMCPMC5372850.
3. Bhatia J. Growth Curves: How to Best Measure Growth of the Preterm Infant. The Journal of Pediatrics. 2013;162(3, Supplement):S2-S6. doi: https://doi.org/10.1016/j.jpeds.2012.11.047.
4. Lau C. Development of infant oral feeding skills: what do we know? The American journal of clinical nutrition. 2016;103(2):616s-21s. Epub 2016/01/23. doi: 10.3945/ajcn.115.109603. PubMed PMID: 26791183; PubMed Central PMCID: PMCPMC4733254.
5. Barlow SM, Finan DS, Lee J, Chu S. Synthetic orocutaneous stimulation entrains preterm infants with feeding difficulties to suck. Journal of perinatology : official journal of the California Perinatal Association. 2008;28(8):541-8. Epub 2008/06/13. doi: 10.1038/jp.2008.57. PubMed PMID: 18548084; PubMed Central PMCID: PMCPMC3725753.
6. Leppänen M, Lapinleimu H, Lind A, Matomäki J, Lehtonen L, Haataja L, et al. Antenatal and postnatal growth and 5-year cognitive outcome in very preterm infants. Pediatrics. 2014;133(1):63-70. Epub 2013/12/18. doi: 10.1542/peds.2013-1187. PubMed PMID: 24344103.
7. Su BH. Optimizing nutrition in preterm infants. Pediatrics and neonatology. 2014;55(1):5-13. Epub 2013/09/21. doi: 10.1016/j.pedneo.2013.07.003. PubMed PMID: 24050843.
8. Bragelien R, Røkke W, Markestad T. Stimulation of sucking and swallowing to promote oral feeding in premature infants. Acta paediatrica (Oslo, Norway : 1992). 2007;96(10):1430-2. Epub 2007/08/24. doi: 10.1111/j.1651-2227.2007.00448.x. PubMed PMID: 17714542.
9. Bingham PM, Ashikaga T, Abbasi S. Prospective study of non-nutritive sucking and feeding skills in premature infants. Archives of disease in childhood Fetal and neonatal edition. 2010;95(3):F194-200. Epub 2009/12/02. doi: 10.1136/adc.2009.164186. PubMed PMID: 19948525.
10. Harding C, Frank L, Van Someren V, Hilari K, Botting N. How does non-nutritive sucking support infant feeding? Infant behavior & development. 2014;37(4):457-64. Epub 2014/06/30. doi: 10.1016/j.infbeh.2014.05.002. PubMed PMID: 24974134.
11. McCain GC. An evidence-based guideline for introducing oral feeding to healthy preterm infants. Neonatal network : NN. 2003;22(5):45-50. Epub 2003/11/06. doi: 10.1891/0730-0832.22.5.45. PubMed PMID: 14598979.
12. Boiron M, Da Nobrega L, Roux S, Henrot A, Saliba E. Effects of oral stimulation and oral support on non-nutritive sucking and feeding performance in preterm infants. Developmental medicine and child neurology. 2007;49(6):439-44. Epub 2007/05/24. doi: 10.1111/j.1469-8749.2007.00439.x. PubMed PMID: 17518930.
13. Foster JP, Psaila K, Patterson T. Non-nutritive sucking for increasing physiologic stability and nutrition in preterm infants. The Cochrane database of systematic reviews. 2016;10(10):Cd001071. Epub 2016/11/02. doi: 10.1002/14651858.CD001071.pub3. PubMed PMID: 27699765; PubMed Central PMCID: PMCPMC6458048.
14. Pinelli J, Symington A. Non-nutritive sucking for promoting physiologic stability and nutrition in preterm infants. The Cochrane database of systematic reviews. 2000;(2):Cd001071. Epub 2000/05/05.

doi: 10.1002/14651858.Cd001071. PubMed PMID: 10796407.

15. Pinelli J, Symington A. Non-nutritive sucking for promoting physiologic stability and nutrition in preterm infants. The Cochrane database of systematic reviews. 2001;(3):Cd001071. Epub 2001/11/01. doi: 10.1002/14651858.Cd001071. PubMed PMID: 11686975.

16. Pinelli J, Symington A. Non-nutritive sucking for promoting physiologic stability and nutrition in preterm infants. The Cochrane database of systematic reviews. 2005;(4):Cd001071. Epub 2005/10/20. doi: 10.1002/14651858.CD001071.pub2. PubMed PMID: 16235279.

17. Liberati A, Altman DG, Tetzlaff J, Mulrow C, Gøtzsche PC, Ioannidis JP, et al. The PRISMA statement for reporting systematic reviews and meta-analyses of studies that evaluate health care interventions: explanation and elaboration. PLoS medicine. 2009;6(7):e1000100. Epub 2009/07/22. doi: 10.1371/journal.pmed.1000100. PubMed PMID: 19621070.

18. Higgins JP, Altman DG, Gøtzsche PC, Jüni P, Moher D, Oxman AD, et al. The Cochrane Collaboration's tool for assessing risk of bias in randomised trials. BMJ (Clinical research ed). 2011;343:d5928. Epub 2011/10/20. doi: 10.1136/bmj.d5928. PubMed PMID: 22008217.

19. Zhang Y, Lyu T, Hu X, Shi P, Cao Y, Latour JM. Effect of nonnutritive sucking and oral stimulation on feeding performance in preterm infants: a randomized controlled trial. Pediatric critical care medicine : a journal of the Society of Critical Care Medicine and the World Federation of Pediatric Intensive and Critical Care Societies. 2014;15(7):608-14. Epub 2014/07/01. doi: 10.1097/pcc.0000000000000182. PubMed PMID: 24977689.

20. Xu DM, Lin M, Huang ZR, Li ZZ, Liang C, Meng Yl, et al. Effects of breast milk olfactory stimulation combined with non-nutritive sucking on feeding outcomes in preterm infants. Journal of Nursing .2022; 37, 28-31.doi:10.3870/j.issn.1001-4152.2022.15.028.

21. Feng JM, Gao L J, Zhang, L. Effects of early swallowing training supplemented with non-nutritive sucking on feeding in very low birth weight preterm infants. Integrative Nursing Care (in Chinese and English) .2022; 8, 82-84.

22. Ling QZ. Effect of non-nutritive sucking on nutrition of 26 preterm infants. Guangxi Medicine .2008;30, 360-361.doi:10.3969/j.issn.0253-4304.2008.03.031.

23. Zhang CY, Xie YP, Zhang CP, Zhu XH, Niu FY. Effect of non-nutritive sucking on the duration of nasogastric tube retention in preterm infants. Journal of Nursing. 2003; 648-649. doi:10.3969/j.issn.1001-4152.2003.09.003.

24. Yue XH, Zhao CX,Lu H, Xue XD. Effects of non-nutritive sucking on nutrition and gastrointestinal transit time in preterm infants. Chinese Journal of Pediatrics. 2003; 15-18. doi:10.3760/j.issn:0578-1310.2003.02.004.

25. Ma LT,Li YL. Study on the effect of non-nutritive sucking combined with mother's voice stimulation intervention in preterm infant feeding. Chinese Journal of Practical Nursing. 2019; 35, 929-932. doi:10.3760/cma.j.issn.1672-7088.2019.12.011.

26. Jiang, GR. Effect of non-nutritive sucking on gastrointestinal feeding in very low birth weight infants. Chinese Journal of Modern Nursing.2009; 15, 2382-2383. doi:10.3760/cma.j.issn.1674-2907.2009.24.013.

27. Kang SQ. Evaluation of the effect of non-nutritive sucking in improving feeding of preterm infants. Chinese Journal of Practical Nursing .2009; 25, 40-41. doi:10.3760/cma.j.issn.1672-7088.2009.03.019.

28. Lyu T-c, Zhang Y-x, Hu X-j, Cao Y, Ren P, Wang Y-j. The effect of an early oral stimulation program on oral feeding of preterm infants. International Journal of Nursing Sciences. 2014;1(1):42-7. doi: https://doi.org/10.1016/j.ijnss.2014.02.010.

29. Lau C, Smith EO. Interventions to improve the oral feeding performance of preterm infants. Acta paediatrica (Oslo, Norway : 1992). 2012;101(7):e269-74. Epub 2012/03/13. doi: 10.1111/j.1651-2227.2012.02662.x. PubMed PMID: 22404221.
30. Field T, Ignatoff E, Stringer S, Brennan J, Greenberg R, Widmayer S, et al. Nonnutritive sucking during tube feedings: effects on preterm neonates in an intensive care unit. Pediatrics. 1982;70(3):381-4. Epub 1982/09/01. PubMed PMID: 6810298.
31. Bernbaum JC, Pereira GR, Watkins JB, Peckham GJ. Nonnutritive sucking during gavage feeding enhances growth and maturation in premature infants. Pediatrics. 1983;71(1):41-5. Epub 1983/01/01. PubMed PMID: 6401358.
32. Shaki F, Aziznejadroshan P, Rad ZA, Chehrazi M, Arzani A. Comparison of the effect of two methods of sucking on pacifier and mother's finger on oral feeding behavior in preterm infants: a randomized clinical trial. BMC pediatrics. 2022;22(1):292. Epub 2022/05/19. doi: 10.1186/s12887-022-03352-9. PubMed PMID: 35585519; PubMed Central PMCID: PMCPMC9118858.
33. Ostadi M, Jokar F, Armanian AM, Namnabati M, Kazemi Y, Poorjavad M. The effects of swallowing exercise and non-nutritive sucking exercise on oral feeding readiness in preterm infants: A randomized controlled trial. International journal of pediatric otorhinolaryngology. 2021;142:110602. Epub 2021/01/08. doi: 10.1016/j.ijporl.2020.110602. PubMed PMID: 33412344.
34. Asadollahpour F, Yadegari F, Soleimani F, Khalesi N. The Effects of Non-Nutritive Sucking and Pre-Feeding Oral Stimulation on Time to Achieve Independent Oral Feeding for Preterm Infants. Iranian journal of pediatrics. 2015;25(3):e809. Epub 2015/07/23. doi: 10.5812/ijp.25(3)2015.809. PubMed PMID: 26199713; PubMed Central PMCID: PMCPMC4505995.
35. Say B, Simsek GK, Canpolat FE, Oguz SS. Effects of Pacifier Use on Transition Time from Gavage to Breastfeeding in Preterm Infants: A Randomized Controlled Trial. Breastfeeding medicine : the official journal of the Academy of Breastfeeding Medicine. 2018;13(6):433-7. Epub 2018/06/19. doi: 10.1089/bfm.2018.0031. PubMed PMID: 29912580.
36. Yildiz A, Arikan D. The effects of giving pacifiers to premature infants and making them listen to lullabies on their transition period for total oral feeding and sucking success. Journal of clinical nursing. 2012;21(5-6):644-56. Epub 2011/06/15. doi: 10.1111/j.1365-2702.2010.03634.x. PubMed PMID: 21668549.
37. Kaya V, Aytekin A. Effects of pacifier use on transition to full breastfeeding and sucking skills in preterm infants: a randomised controlled trial. Journal of clinical nursing. 2017;26(13-14):2055-63. Epub 2016/10/19. doi: 10.1111/jocn.13617. PubMed PMID: 27754572.
38. Fucile S, Milutinov M, Timmons K, Dow K. Oral Sensorimotor Intervention Enhances Breastfeeding Establishment in Preterm Infants. Breastfeeding medicine : the official journal of the Academy of Breastfeeding Medicine. 2018;13(7):473-8. Epub 2018/08/17. doi: 10.1089/bfm.2018.0014. PubMed PMID: 30113209.
39. Rocha AD, Moreira ME, Pimenta HP, Ramos JR, Lucena SL. A randomized study of the efficacy of sensory-motor-oral stimulation and non-nutritive sucking in very low birthweight infant. Early human development. 2007;83(6):385-8. Epub 2006/09/19. doi: 10.1016/j.earlhumdev.2006.08.003. PubMed PMID: 16979854.
40. Morag I, Hendel Y, Karol D, Geva R, Tzipi S. Transition From Nasogastric Tube to Oral Feeding: The Role of Parental Guided Responsive Feeding. Frontiers in pediatrics. 2019;7:190. Epub 2019/05/31. doi: 10.3389/fped.2019.00190. PubMed PMID: 31143759; PubMed Central PMCID: PMCPMC6521795.
41. Khodagholi Z, Zarifian T, Soleimani F, Khoshnood Shariati M, Bakhshi E. The Effect of Non-Nutritive

Sucking and Maternal Milk Odor on the Independent Oral Feeding in Preterm Infants. Iranian journal of child neurology. 2018;12(4):55-64. Epub 2018/10/04. PubMed PMID: 30279709; PubMed Central PMCID: PMCPMC6160630.

42. Barlow SM, Finan DS, Rowland SG. Mechanically evoked perioral reflexes in infants. Brain research. 1992;599(1):158-60. Epub 1992/12/18. doi: 10.1016/0006-8993(92)90865-7. PubMed PMID: 1493544.

43. Liao C, Rosner AO, Maron JL, Song D, Barlow SM. Automatic Nonnutritive Suck Waveform Discrimination and Feature Extraction in Preterm Infants. Computational and mathematical methods in medicine. 2019;2019:7496591. Epub 2019/03/14. doi: 10.1155/2019/7496591. PubMed PMID: 30863456; PubMed Central PMCID: PMCPMC6378788.

44. Tolppola O, Renko M, Sankilampi U, Kiviranta P, Hintikka L, Kuitunen I. Pacifier use and breastfeeding in term and preterm newborns-a systematic review and meta-analysis. European journal of pediatrics. 2022;181(9):3421-8. Epub 2022/07/15. doi: 10.1007/s00431-022-04559-9. PubMed PMID: 35834044; PubMed Central PMCID: PMCPMC9395499.

45. Schwartz R, Moody L, Yarandi H, Anderson GC. A meta-analysis of critical outcome variables in nonnutritive sucking in preterm infants. Nursing Research. 1987;36(5):292-5. doi: 10.1097/00006199-198709000-00010.

46. Marchini G, Lagercrantz H, Feuerberg Y, Winberg J, Uvnäs-Moberg K. The effect of non-nutritive sucking on plasma insulin, gastrin, and somatostatin levels in infants. Acta paediatrica Scandinavica. 1987;76(4):573-8. Epub 1987/07/01. doi: 10.1111/j.1651-2227.1987.tb10523.x. PubMed PMID: 2888258.

47. Chorna OD, Slaughter JC, Wang L, Stark AR, Maitre NL. A pacifier-activated music player with mother's voice improves oral feeding in preterm infants. Pediatrics. 2014;133(3):462-8. Epub 2014/02/19. doi: 10.1542/peds.2013-2547. PubMed PMID: 24534413; PubMed Central PMCID: PMCPMC3934339.

48. Calik C, Esenay F. The clinical effect of pacifier use on orogastric tube-fed preterm infants: A randomized controlled trial. JPMA The Journal of the Pakistan Medical Association. 2019;69(6):771-6. Epub 2019/06/14. PubMed PMID: 31189280.

49. Widström AM, Marchini G, Matthiesen AS, Werner S, Winberg J, Uvnäs-Moberg K. Nonnutritive sucking in tube-fed preterm infants: effects on gastric motility and gastric contents of somatostatin. Journal of pediatric gastroenterology and nutrition. 1988;7(4):517-23. Epub 1988/07/01. doi: 10.1097/00005176-198807000-00007. PubMed PMID: 2899621.

50. Hu QY, Qin XL, Cao P, Sun Y, Wei YH. Effect of feeding continuity of care on growth and development of preterm infants at nutritional risk. Journal of Nursing .2020; 35, 101-104. doi:10.3870/j.issn.1001-4152.2020.21.101.

51. Dong YH, Yang XC, Zhang JP,Yang FT, Qin JX, You XL,et al. Developmental supportive care in the intensive care unit of preterm infants. Chinese Journal of Practical Nursing. 2007; 23, 29-30. doi:10.3760/cma.j.issn.1672-7088.2007.13.014.

52. Premji SS, Paes B. Gastrointestinal function and growth in premature infants: is non-nutritive sucking vital? Journal of perinatology : official journal of the California Perinatal Association. 2000;20(1):46-53. Epub 2000/02/29. doi: 10.1038/sj.jp.7200299. PubMed PMID: 10693100.

53. Ma L., Tao YQ. Research Progress of Oral Motor Intervention to Improve Transoral Feeding in Preterm Infants. Chinese Journal of Modern Nursing .2015; (35):4332-4,5. doi:10.3760/cma.j.issn.1674-2907.2015.35.036.

Table 1. Characteristics of the included studies.

Study	Year	Country	Number		Intervention		Outcomes
			NNS	Control	NNS group	Control group	
Shaki	2022	Iran	50	50	Sucking on a pacifier 3 times/day followed by gavage for ten days of intervention.	routine ward care	①②③
Ostadi	2021	Iran.	15	15	provided with NNS twice a day, for 10 days during two consecutive weeks	not provided with any intervention	①②③
Say	2018	Turkey	45	45	Use the soother for 15 minutes before and after feeding, 4 times/day.	not provided with any intervention	①②④⑥⑦
Fucile	2018	Canada	16	15	The intervention was a 15-minute program consisting of 5 minutes of preparatory oral stimulation of the cheeks and lips, 5 minutes of tongue exercises, and 5 minutes of NNS. 1x/day for 10 days.	15 minutes of pseudo-intervention	①②⑤
Vildan	2017	Turkey	34	36	Sucking 3 times a day to soothe	Not using soothers	②③④⑤
Asadollahpour	2015	Iran	11	11	NNS 3 times/day for 15 minutes each time. Place little finger in infant's mouth and stroke palate for 5 min/times to elicit sucking.	pseudo-intervention	①②④
Zhang	2014	China	25	27	Suck on the pacifier 7-8 times/day for 5 minutes.	not provided with any intervention	①②③⑤
Yildiz	2012	Turkey	30	30	3x/day sucking on a pacifier until oral feeding is initiated	Routine gavage feeding without NNS	①②④
Rocha	2007	Brazil	49	49	NNS 15min/dose for 10 days until oral feeding is initiated	15 minutes of pseudo-intervention	①②③④⑤
Lau	2012	USA	25	23	NNS, 15 minutes/day, 5 days per week.	Routine feeding care	①②③
Field	1982	USA	30	27	infants in the treatment group were given a pacifier during all tube feedings.	Control infants were tube-fed only until oral feeding	②③
Bernbaum	1983	USA	15	15	Infants in the intervention group were given rubber nipples for NNS during the entire feeding process	Control infants were tube-fed only until oral feeding	①②
Xu	2022	China	57	57	Breast milk olfactory stimulation combined with NNS.	Routine feeding care	②③④⑦
Feng	2022	China	44	46	Early swallowing function training combined with NNS	Routine feeding care	①③⑦
Ling	2008	China	26	21	NNS was given before each nasogastric tube feeding and sucked for 10min every 2-3h/time.	No NNS	②③⑥⑦
Zhang	2003	China	22	21	NNS every 3h with 10min sucking for 2 weeks.	No NNS	②③⑥
Yue	2003	China	18	20	Preterm infants were given a non-porous rubber nipple to suck for 5 min before, during and after each nasogastric tube feeding for 2 weeks.	No NNS	③⑥⑦

Ma	2019	China	43	43	NNS was given for 5 min before each feeding until the preterm infant was able to complete 8 sucks/d for 3 h/suck for 7 d of continuous intervention.	No NNS	①②
Jiang	2009	China	50	48	Preterm infants were given a non-porous rubber nipple to suck for 5 min before, during and after each feeding, 7-8 times/24h, for a period of 1 week.	Routine gavage feeding without NNS	③⑥⑦
Kang	2009	China	36	36	Sucking on a non-porous rubber nipple for 10 min before each feeding, 8 times/d.	Routine gavage feeding without NNS	③⑥⑦
Lyu	2014	China	32	31	Implemented an oral stimulation program consisting of 12 minutes of oral stimulation and 3 minutes of NNS. administered 15-30 minutes before feeding, once a day.	Routine feeding care in the NICU	①④⑤⑥

Note:①Time taken to achieve exclusive oral feeding(days) ②Length of hospital stay(days) ③Time to start oral feeding（days) ④Body weight of discharge（g）

⑤Weight at full oral feeding (g) ⑥Time to return to birth weight(days) ⑦Gastrointestinal complications

Table 2. Results of sensitivity analysis.

Outcome indicator	Fixed effect model *OR*（*95%CI*）	random effect model *OR*（*95%CI*）
Time taken to achieve exclusive oral feeding	-4.19 (-4.73, -3.64)	-4.65（-6.11,-3.20）
Length of hospital stay	-3.56（-4.29,-2.82）	-4.58（-6.40,-2.76）
Time to start oral feeding	-1.39（-1.88,-0.90）	-1.41 (-2.36, -0.45)
Weight of discharge	-63.48（-97.93,-29.03）	-63.95（-99.29,-28.62）

Weight at full oral feeding	-92.91（-142.68,-43.13）	-92.91（-142.68,-43.13）
Time to return to birth weight	-1.67（-2.17,-1.17）	-1.72 (-2.54, -0.91)
Gastrointestinal complications	0.22 (0.14,0.35)	0.22 (0.14,0.35)

Figure legends:

Fig1. PRISMA flow diagram.

Fig2. Assessment of risk of bias. **Fig2. (a):** Risk of bias graph about each risk of bias item presented as percentages across all

included studies. **Fig2. (b):** Summary of risk of bias for each trial.

Fig3. Forest plot for time taken to achieve exclusive oral feeding.

Fig4. Forest plot for length of hospital stay.

Fig5. Forest plot for time to start oral feeding.

Fig6. Forest plot for weight of discharge.

Fig7. Forest plot for time to return to birth weight.

Fig8. Forest plot for weight at full oral feeding.

Fig9. Forest plot for gastrointestinal complications.

Fig1.

Click here to access/download;Figure;Fig1..tif

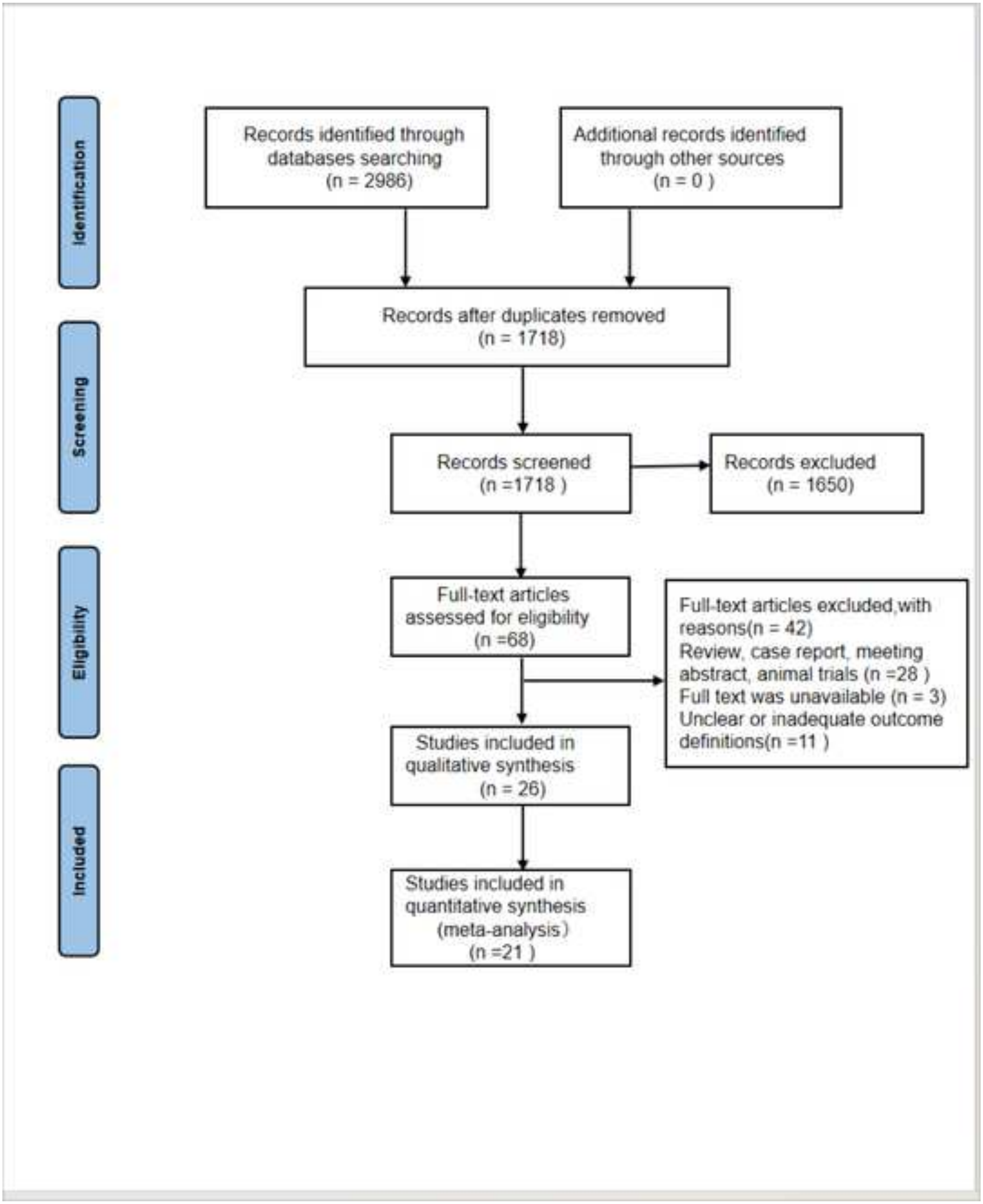

Fig.2

Click here to access/download;Figure;Fig2.(a).tif

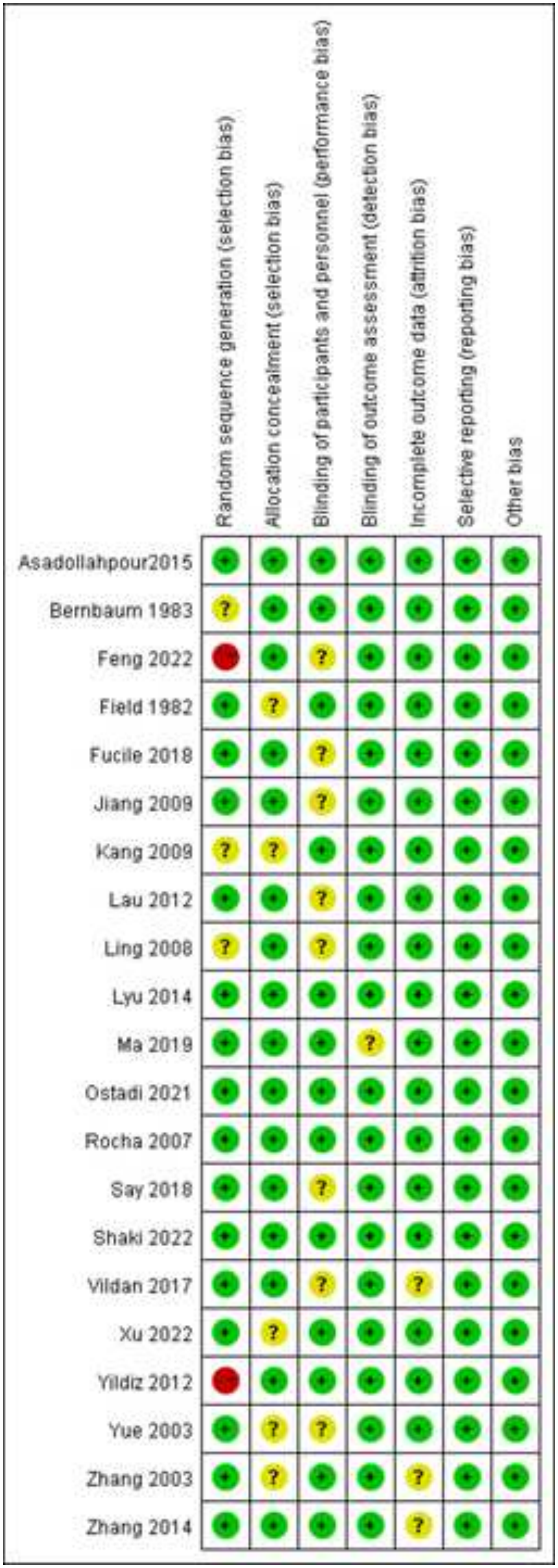

Fig.3

Click here to access/download;Figure;Fig2.(b).tif

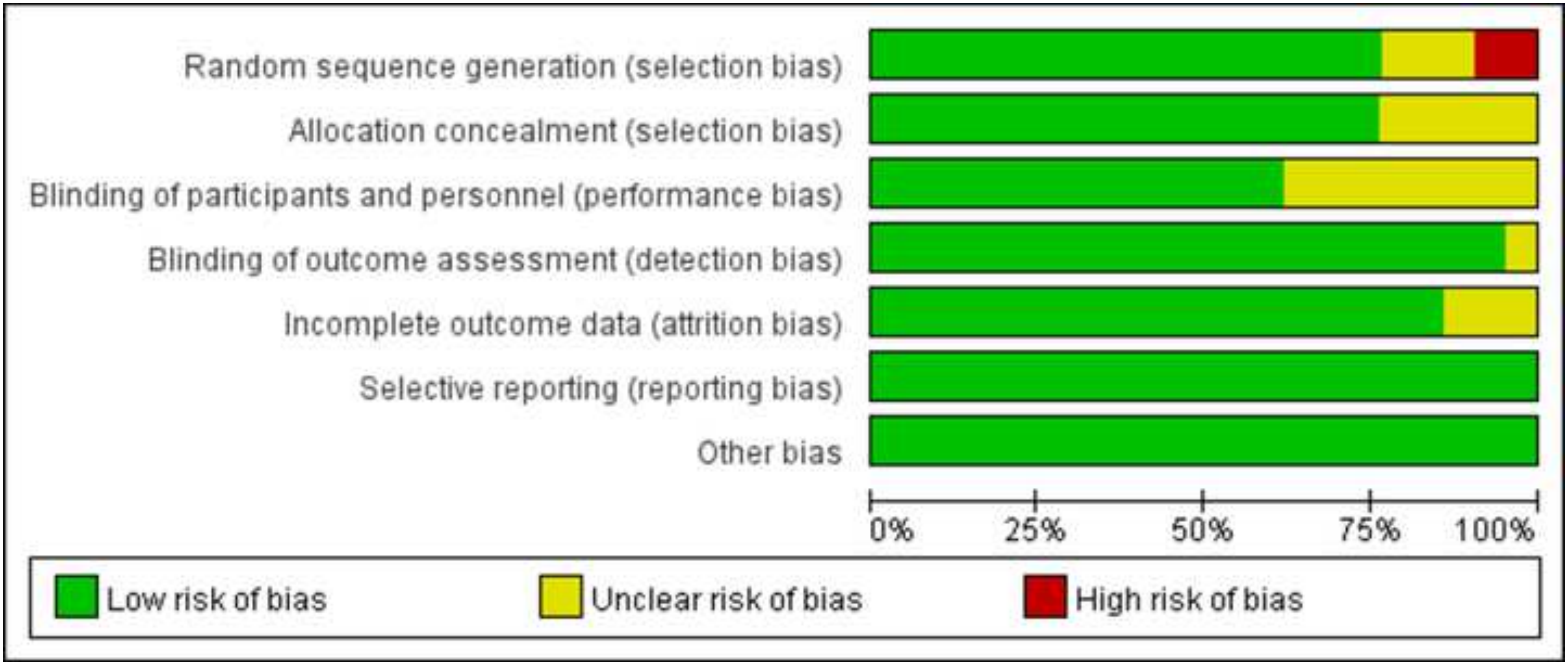

Fig.4

Click here to access/download;Figure;Fig3..tif

	NNS			Control				Mean Difference
Study or Subgroup	Mean	SD	Total	Mean	SD	Total	Weight	IV, Random, 95% CI
Asadollahpour2015	16.72	8.94	11	24.27	9.42	11	2.8%	-7.55 [-15.22, 0.12]
Bernbaum 1983	10.4	2.1	15	16.1	5.1	15	8.5%	-5.70 [-8.49, -2.91]
Feng 2022	26.18	9.94	44	30.87	10.92	46	5.9%	-4.69 [-9.00, -0.38]
Fucile 2018	10.7	2.1	16	19.3	3.6	15	9.8%	-8.60 [-10.69, -6.51]
Lau 2012	18.9	1.7	25	20.8	1.9	23	11.6%	-1.90 [-2.92, -0.88]
Lyu 2014	9.56	4.43	32	13.19	6.18	31	8.7%	-3.63 [-6.29, -0.97]
Ma 2019	12.09	2.1	43	17.7	2.3	43	11.7%	-5.61 [-6.54, -4.68]
Ostadi 2021	8.6	7.1	13	12.4	6.2	13	4.9%	-3.80 [-8.92, 1.32]
Rocha 2007	38.5	16.4	49	47.2	17.7	49	3.4%	-8.70 [-15.46, -1.94]
Say 2018	38.1	20	45	49.1	22	45	2.3%	-11.00 [-19.69, -2.31]
Shaki 2022	22.54	7.54	50	25.86	7.93	50	8.0%	-3.32 [-6.35, -0.29]
Xu 2022	15	5.19	57	17	4.44	57	10.4%	-2.00 [-3.77, -0.23]
Yildiz 2012	7.68	3.26	30	11.68	7.29	30	8.3%	-4.00 [-6.86, -1.14]
Zhang 2014	27	10.5	25	30	11.8	27	3.9%	-3.00 [-9.06, 3.06]
**Total (95% CI)**			**455**			**455**	**100.0%**	**-4.65 [-6.11, -3.20]**

Heterogeneity: $Tau^2 = 4.51$; $Chi^2 = 57.76$, df = 13 (P < 0.00001); $I^2 = 77\%$

Test for overall effect: Z = 6.26 (P < 0.00001)

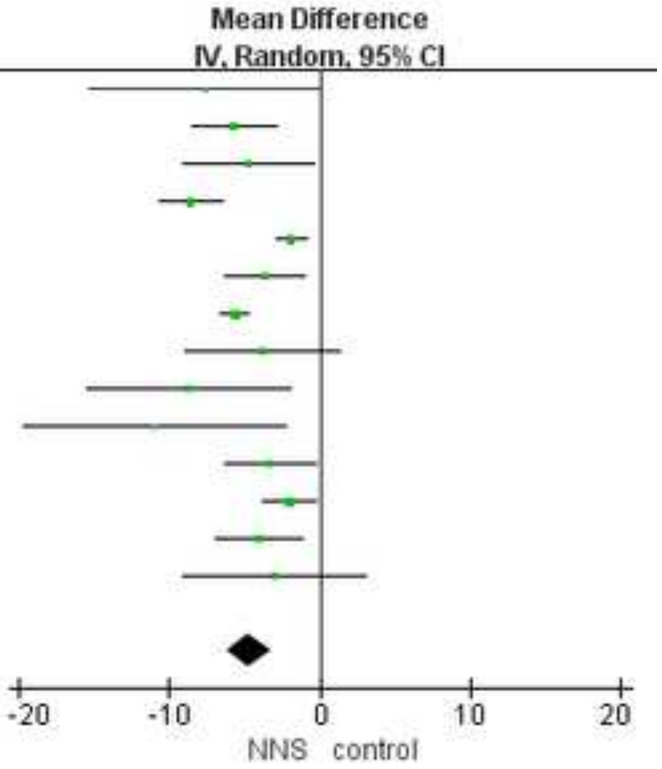

Click here to access/download;Figure;Fig4..tif

Study or Subgroup	NNS Mean	NNS SD	NNS Total	Control Mean	Control SD	Control Total	Weight	Mean Difference IV, Random, 95% CI
**1.2.2 gestational age >33 weeks**								
Bernbaum 1983	51.9	8.2	15	58.7	9.8	15	4.7%	-6.80 [-13.27, -0.33]
Field 1982	48	21	30	56	18	27	2.5%	-8.00 [-18.13, 2.13]
Fucile 2018	33	15.7	16	43.2	27.3	15	1.2%	-10.20 [-26.01, 5.61]
Ling 2008	18.29	4.62	26	25.23	3.84	21	9.5%	-6.94 [-9.36, -4.52]
Ma 2019	14.7	1.79	43	19.81	4.17	43	10.8%	-5.11 [-6.47, -3.75]
Vildan 2017	18.1	5.55	34	24.73	16.06	36	5.5%	-6.63 [-12.20, -1.06]
Xu 2022	25	8.89	57	31	13.33	57	7.2%	-6.00 [-10.16, -1.84]
Yildiz 2012	15.37	4.94	30	21.75	11.24	30	6.9%	-6.38 [-10.77, -1.99]
Zhang 2014	38	13.9	25	41.4	12.9	27	4.0%	-3.40 [-10.71, 3.91]
**Subtotal (95% CI)**			**276**			**271**	**52.3%**	**-5.70 [-6.74, -4.65]**
Heterogeneity: Tau^2 = 0.00; Chi^2 = 2.96, df = 8 (P = 0.94); I^2 = 0%								
Test for overall effect: Z = 10.67 (P < 0.00001)								
**1.2.3 gestational age ≤33 weeks**								
Asadollahpour2015	26.54	12.48	11	33.45	13.46	11	2.3%	-6.91 [-17.76, 3.94]
Lau 2012	76.2	5.6	25	75	4.7	23	8.9%	1.20 [-1.72, 4.12]
Ostadi 2021	33.7	2.7	13	33.4	1.7	13	10.4%	0.30 [-1.43, 2.03]
Rocha 2007	41.9	17.4	49	52.3	19.5	49	4.0%	-10.40 [-17.72, -3.08]
Say 2018	48.4	19.2	45	65.3	30.6	45	2.4%	-16.90 [-27.45, -6.35]
Shaki 2022	27.28	6.2	50	29.36	5.97	50	9.6%	-2.08 [-4.47, 0.31]
Zhang 2003	21.47	3.14	22	24.56	3.22	21	10.2%	-3.09 [-4.99, -1.19]
**Subtotal (95% CI)**			**215**			**212**	**47.7%**	**-2.80 [-5.39, -0.20]**
Heterogeneity: Tau^2 = 7.22; Chi^2 = 25.15, df = 6 (P = 0.0003); I^2 = 76%								
Test for overall effect: Z = 2.11 (P = 0.03)								
**Total (95% CI)**			**491**			**483**	**100.0%**	**-4.58 [-6.40, -2.76]**
Heterogeneity: Tau^2 = 7.54; Chi^2 = 59.80, df = 15 (P < 0.00001); I^2 = 75%								
Test for overall effect: Z = 4.93 (P < 0.00001)								
Test for subgroup differences: Chi^2 = 4.12, df = 1 (P = 0.04), I^2 = 75.7%								

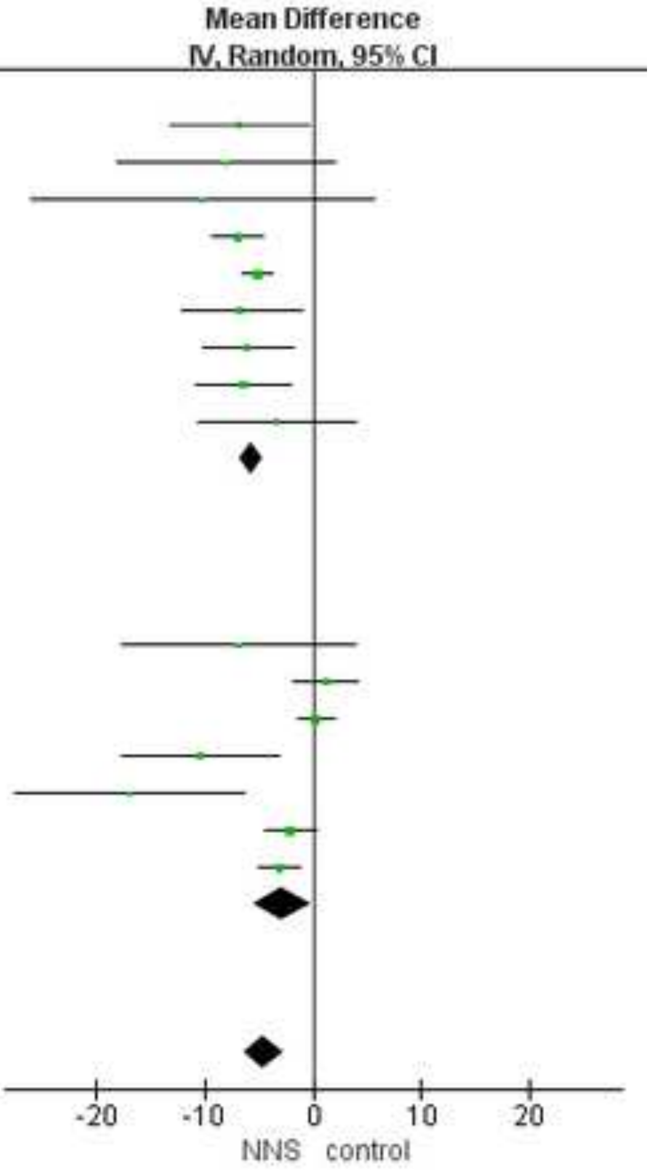

Fig.6

Click here to access/download;Figure;Fig5..tif

Study or Subgroup	NNS Mean	NNS SD	NNS Total	Control Mean	Control SD	Control Total	Weight	Mean Difference IV, Random, 95% CI
Feng 2022	12.81	3.63	44	14.78	4.71	46	10.8%	-1.97 [-3.70, -0.24]
Field 1982	26	21	30	29	18	27	0.8%	-3.00 [-13.13, 7.13]
Jiang 2009	5.1	2.3	50	6.6	2.5	48	14.5%	-1.50 [-2.45, -0.55]
Kang 2009	12.3	4.1	36	15.6	4.5	36	9.7%	-3.30 [-5.29, -1.31]
Lau 2012	44.4	4.9	25	41.7	3.6	23	8.1%	2.70 [0.28, 5.12]
Ling 2008	13	9	26	20	12	21	2.1%	-7.00 [-13.19, -0.81]
Ostadi 2021	32.1	1.3	13	32.7	1.4	13	14.1%	-0.60 [-1.64, 0.44]
Rocha 2007	32.3	15.4	49	40.5	16.3	49	2.0%	-8.20 [-14.48, -1.92]
Shaki 2022	12.52	4.52	50	13.14	5.17	50	10.1%	-0.62 [-2.52, 1.28]
Vildan 2017	12.56	4.63	34	14.94	10.41	36	4.7%	-2.38 [-6.12, 1.36]
Xu 2022	15	10.37	57	14	10.37	57	4.6%	1.00 [-2.81, 4.81]
Yue 2003	13	10	18	17	12	20	1.7%	-4.00 [-11.00, 3.00]
Zhang 2003	14.23	2.19	22	16.43	1.46	21	13.8%	-2.20 [-3.31, -1.09]
Zhang 2014	17	10	25	15.5	7.8	27	3.1%	1.50 [-3.40, 6.40]
**Total (95% CI)**			**479**			**474**	**100.0%**	**-1.41 [-2.36, -0.45]**

Heterogeneity: $Tau^2 = 1.40$; $Chi^2 = 31.33$, df = 13 (P = 0.003); $I^2 = 59\%$

Test for overall effect: Z = 2.89 (P = 0.004)

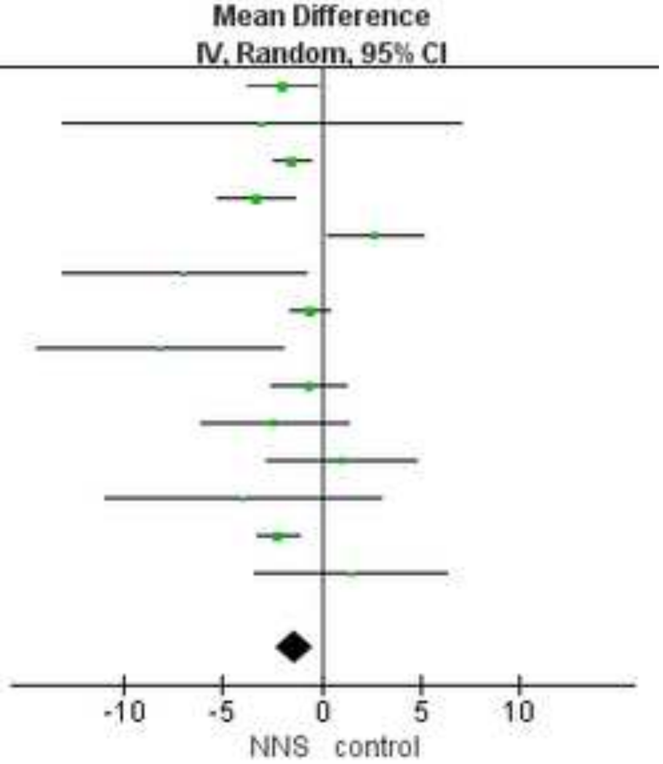

Fig.7

Click here to access/download;Figure;Fig6..tif

	NNS			Control				Mean Difference
Study or Subgroup	Mean	SD	Total	Mean	SD	Total	Weight	IV, Fixed, 95% CI
Lau 2012	2,807	105	25	2,849	104	23	33.9%	-42.00 [-101.17, 17.17]
Lyu 2014	2,086.56	115.24	32	2,178.39	210.02	31	16.8%	-91.83 [-175.85, -7.81]
Rocha 2007	1,901	237	49	1,997	327	49	9.3%	-96.00 [-209.08, 17.08]
Say 2018	2,099.6	181.6	45	2,134.2	491.9	45	5.1%	-34.60 [-187.80, 118.60]
Vildan 2017	1,956.45	268.04	34	2,159.75	341.22	36	5.8%	-203.30 [-346.62, -59.98]
Xu 2022	2,210	290	57	2,220	240	57	12.4%	-10.00 [-107.72, 87.72]
Yildiz 2012	1,803	146.62	30	1,863.67	184.1	30	16.7%	-60.67 [-144.89, 23.55]
**Total (95% CI)**			**272**			**271**	**100.0%**	**-63.48 [-97.93, -29.03]**

Heterogeneity: $Chi^2 = 6.21$, $df = 6$ ($P = 0.40$); $I^2 = 3\%$

Test for overall effect: $Z = 3.61$ ($P = 0.0003$)

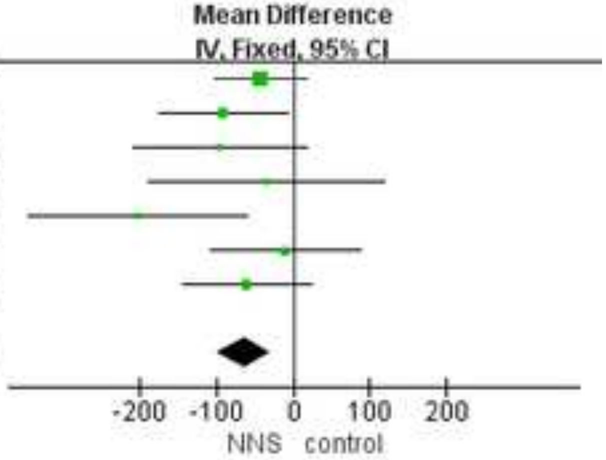

Fig.8

Click here to access/download;Figure;Fig7..tif

	NNS			Control				Mean Difference
Study or Subgroup	Mean	SD	Total	Mean	SD	Total	Weight	IV, Random, 95% CI
Jiang 2009	12.9	2.7	50	16	3.3	48	18.4%	-3.10 [-4.30, -1.90]
Kang 2009	9.2	2.4	36	11.5	3.5	36	16.2%	-2.30 [-3.69, -0.91]
Ling 2008	8.9	3.72	26	9.14	2.56	21	12.2%	-0.24 [-2.04, 1.56]
Lyu 2014	13.61	5.04	32	13.87	4.29	31	8.8%	-0.26 [-2.57, 2.05]
Say 2018	11.7	4	45	13.8	5.6	45	10.6%	-2.10 [-4.11, -0.09]
Yue 2003	8.8	3.7	18	11.1	3	20	9.7%	-2.30 [-4.46, -0.14]
Zhang 2003	8.76	0.83	22	9.94	1.56	21	24.2%	-1.18 [-1.93, -0.43]
**Total (95% CI)**			**229**			**222**	**100.0%**	**-1.72 [-2.54, -0.91]**

Heterogeneity: $Tau^2 = 0.56$; $Chi^2 = 12.27$, df = 6 (P = 0.06); $I^2 = 51\%$

Test for overall effect: Z = 4.16 (P < 0.0001)

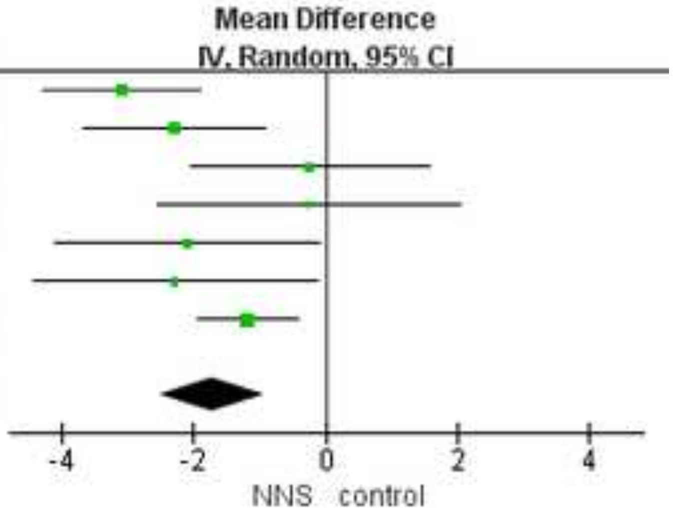

Fig.9

Click here to access/download;Figure;Fig8..tif

	NNS			Control				Mean Difference
Study or Subgroup	Mean	SD	Total	Mean	SD	Total	Weight	IV, Fixed, 95% CI
Fucile 2018	2,331	216	16	2,443	572	15	2.6%	-112.00 [-420.21, 196.21]
Lyu 2014	2,086.56	115.24	32	2,178.39	210.02	31	35.1%	-91.83 [-175.85, -7.81]
Rocha 2007	1,819	216	49	1,882	275	49	25.8%	-63.00 [-160.91, 34.91]
Vildan 2017	1,874.5	279.74	34	1,970.14	309.96	36	13.0%	-95.64 [-233.82, 42.54]
Zhang 2014	1,853.2	165.2	25	1,977	211.4	27	23.5%	-123.80 [-226.52, -21.08]
**Total (95% CI)**			**156**			**158**	**100.0%**	**-92.91 [-142.68, -43.13]**

Heterogeneity: $Chi^2 = 0.72$, $df = 4$ ($P = 0.95$); $I^2 = 0\%$

Test for overall effect: $Z = 3.66$ ($P = 0.0003$)

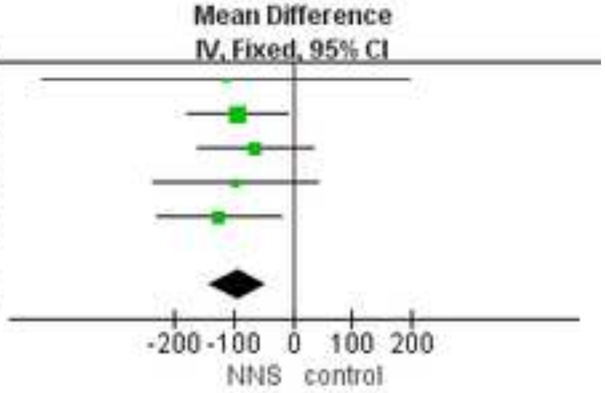

Fig.10

Click here to access/download;Figure;Fig9..tif

Study or Subgroup	NNS Events	NNS Total	Control Events	Control Total	Weight	Odds Ratio M-H, Fixed, 95% CI
Feng 2022	2	44	10	46	10.3%	0.17 [0.04, 0.83]
Jiang 2009	9	50	21	48	19.4%	0.28 [0.11, 0.71]
Kang 2009	12	36	28	36	20.7%	0.14 [0.05, 0.41]
Ling 2008	10	26	14	21	10.5%	0.31 [0.09, 1.04]
Say 2018	6	45	21	45	20.1%	0.18 [0.06, 0.50]
Xu 2022	4	57	9	57	9.3%	0.40 [0.12, 1.39]
Yue 2003	3	18	11	20	9.6%	0.16 [0.04, 0.75]
**Total (95% CI)**		**276**		**273**	**100.0%**	**0.22 [0.14, 0.35]**
Total events	46		114			

Heterogeneity: $Chi^2 = 2.58$, $df = 6$ ($P = 0.86$); $I^2 = 0\%$

Test for overall effect: $Z = 6.73$ ($P < 0.00001$)

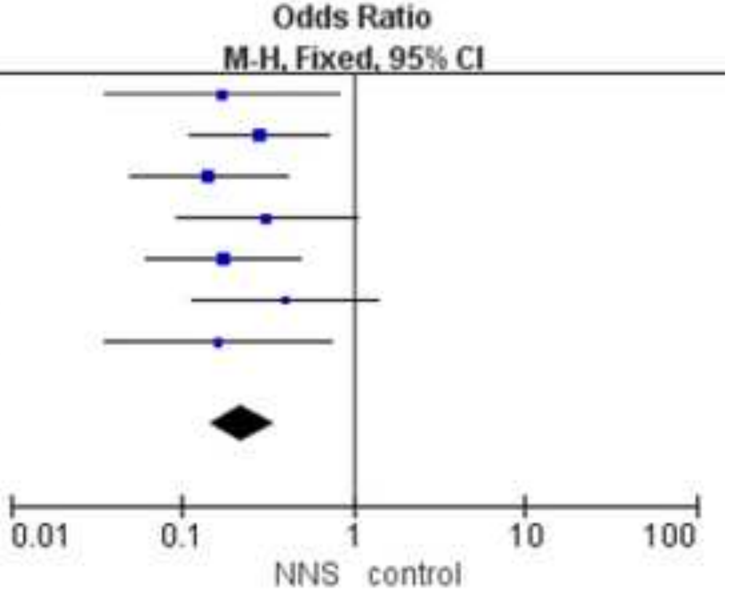

Click here to access/download
Supporting Information
S1 Table. PRISMA2020checklist.docx

Click here to access/download
Supporting Information
S2 Table..docx

Click here to access/download

Supporting Information - Compressed/ZIP File Archive

S1 Fig..zip
