# Supplementary material for: Effects of implementing non-nutritive sucking on oral feeding progression and outcomes in preterm infants: A systematic review and meta-analysis
Source: PLoS One. 2024 Apr 16;19(4):e0302267. doi: 10.1371/journal.pone.0302267 (PMC11020483; doi:10.1371/journal.pone.0302267)
Supplement: Attachment — Submitted filename: Report.docx [file pone.0302267.s005.docx]

**PONE-D-23-30243**

**Effectiveness of non-nutritive sucking on oral feeding of preterm infants: a systematic review and meta-analysis**

**PLOS ONE**

**Thank you for asking me to review this study.**

1. Please adjust the keywords according to the mesh.
2. The most important criteria for entering and exiting the study should be mentioned in the method section of the abstract.
3. Please add the search thermals key words in the method section of the abstract if there is no word limit.
4. The time period of the search (from the beginning to the end) should be fully stated.
5. The title or research question of a systematic review should be designed based on PICO, so the comparison group, which is the same as the control group, should be added in the title.

Thank you
