# Supplementary material for: Effects of implementing non-nutritive sucking on oral feeding progression and outcomes in preterm infants: A systematic review and meta-analysis
Source: PLoS One. 2024 Apr 16;19(4):e0302267. doi: 10.1371/journal.pone.0302267 (PMC11020483; doi:10.1371/journal.pone.0302267)
Supplement: Attachment — Submitted filename: Report - 2.docx [file pone.0302267.s007.docx]

**PONE-D-23-30243**

**Effectiveness of non-nutritive sucking on oral feeding of preterm infants: a systematic review and meta-analysis**

**PLOS ONE**

**Thank you for asking me to review this study.** **The authors' answers were acceptable to me and convincing.**

Thank you
