# Supplementary material for: Effects of implementing non-nutritive sucking on oral feeding progression and outcomes in preterm infants: A systematic review and meta-analysis
Source: PLoS One. 2024 Apr 16;19(4):e0302267. doi: 10.1371/journal.pone.0302267 (PMC11020483; doi:10.1371/journal.pone.0302267)
Supplement: Attachment — Submitted filename: Response to Reviewers.docx [file pone.0302267.s008.docx]

*PLOS ONE* **PONE-D-23-30243R1** Effects of non-nutritive sucking on developmental progress and oral feeding outcomes in preterm infants: a systematic review and meta-analysis

**Response to the Reviewers' Concerns**

**Reviewer #1:**  Review 16/3/2024

Thank you for your extensive work and addressing the suggestions and comments. The manuscript demonstrated thorough and diligent efforts

Kindly find below minor suggestions for your kind follow up best of luck

Response: Once again, we express our appreciation for your constructive comments. Here, we have responded to the comments individually in blue.

1- Abstract:

a. Title: Based on your research question you may need to modify the title as: “Effects Of Implementing Non-Nutritive Sucking On Oral Feeding Progression And Outcomes In Preterm Infants: A Systematic Review And Meta-Analysis”

b. Aim: Line 34-35 You may need to review the aim “to explore the effect of NNS on oral feeding progression through a meta-analysis”.

c. Conclusion: Line 64 you may need to modify the sentence to “reduces the time to reach full oral feeding”

Response: Thanks to the reviewer's comment.

a. As per your suggestion, I have revised the title to " Effects Of Implementing Non-Nutritive Sucking On Oral Feeding Progression And Outcomes In Preterm Infants: A Systematic Review And Meta-Analysis."
b. The purpose of the study in the abstract section has been revised to: "This review aimed to explore the effect of NNS on oral feeding progression through a meta-analysis. "
c. The first sentence of the conclusion has been modified as per your suggestion to read " NNS improves oral feeding outcomes in preterm infants and reduces the time to reach full oral feeding and hospitalization length."

2- Introduction: Line 88-90 the cited reference discusses the effect of oxygen therapy on altering the oral sensory, motor, and the development of coordinated NNS in preterm infants. I may suggest reviewing another reference to support your point.

Response: We have added other references to support our argument.

(9.	Samara M, Johnson S, Lamberts K, Marlow N, Wolke D. Eating problems at age 6 years in a whole population sample of extremely preterm children. Developmental medicine and child neurology. 2010;52(2):e16-22. Epub 2009/10/17. doi: 10.1111/j.1469-8749.2009.03512.x. PubMed PMID: 19832883.)

3- Materials and methods

a. Systematic search and strategy

Line 143 -1 44 “Detailed search strategies for each database are shown in S2 Table” you may need to modify to S1 table as stated in the section of supporting information.

b. Inclusion and exclusion criteria: Line 157-165: You may need to put references for the definition of outcome measures and add the full word of the unit “g” as gram

Response: Thanks to the reviewer's comment.

a. See S1 Table for detailed search strategies for each database, which we have modified.

b. We have added references to all the definitions for the outcome measures and modified the unit "g" to "gram". (lines 159-168)

4- Results: Line 215 - 227: Please review the values in the related section and match with ones stated in Fig1. PRISMA flow diagram

Response: Lines 216-228, we checked this section well compared to the values in Figure 1 (PRISMA flow diagram) and verified that there were no errors.

5- Discussion:

a. Line 325, please review the citation, it is more pertinent for the reference (57)

“Barlow SM, Finan DS, Rowland SG. Mechanically evoked perioral reflexes in infants. Brain research. 651 1992;599(1):158-60. Epub 1992/12/18. doi: 10.1016/0006-8993(92)90865-7. PubMed PMID: 1493544.”

Response: We amend the citation in line 325 to read "59.	Barlow SM, Finan DS, Rowland SG. Mechanically evoked perioral reflexes in infants. Brain research. 1992;599(1):158-60. Epub 1992/12/18. doi: 10.1016/0006-8993(92)90865-7. PubMed PMID: 1493544."

b. Line 347, The author Chorna et al studied the outcome for the development of sucking ability and decrease the length of hospitalization. I may suggest looking at this reference instead which elaborates the complex factors for discharge “Committee on Fetus and Newborn. (2008). Hospital discharge of the high-risk neonate. Pediatrics, 122(5), 1119-1126.”

Response: As you suggested, we have carefully read and quoted from the article (62. Hospital discharge of the high-risk neonate. Pediatrics. 2008;122(5):1119-26. Epub 2008/11/04. doi: 10.1542/peds.2008-2174. PubMed PMID: 18977994.) and have refined this section of the manuscript. (lines 346-352)

**Reviewer #2:** Thank you for asking me to review this study. The authors' answers were acceptable to me and convincing.

Response: We are pleased to learn that you found our response acceptable and convincing. Thank you again for your interest in and support of our research, and good luck.

**We would like to thank the reviewer again for your time and efforts to provide the comments for us to improve the manuscript.**
